# Chirality imprinting and direct asymmetric reaction screening using a stereodynamic Brønsted/Lewis acid receptor

**DOI:** 10.1038/ncomms12539

**Published:** 2016-08-23

**Authors:** Keith W. Bentley, Daysi Proano, Christian Wolf

**Affiliations:** 1 Department of Chemistry, Georgetown University, Washington, DC 20057, USA

## Abstract

Molecular recognition, activation and dynamic self-assembly with Brønsted and Lewis acids play a central role across the chemical sciences including catalysis, crystal engineering, supramolecular architectures and drug design. Despite this general advance, the utilization of the corresponding binding motifs for fast and robust quantitative chemosensing of chiral compounds in a complicate matrix has remained challenging. Here we show that a stereodynamic probe carrying complementary boronic acid and urea units achieves this goal with hydroxy carboxylic acids. Synergistic dual-site binding and instantaneous chirality imprinting result in characteristic ultraviolet and CD readouts that allow instantaneous determination of the absolute configuration, enantiomeric excess and concentration of the target compound even in complex mixtures. The robustness and practicality of this strategy for high-throughput screening purposes is demonstrated. Comprehensive sensing of only 0.5 mg of a crude reaction mixture of an asymmetric reduction eliminates cumbersome work-up protocols and minimizes analysis time, labour and waste production.

## Abstract (web summary)

Determining results of asymmetric reactions can take long periods of time and consume large amounts of organic solvents during work-up and analysis. Here, the authors report a bifunctional organic probe that can bind to chiral hydroxyacids, and provide yield, enantiomeric excess and absolute configuration even with crude mixtures.

## Graphical abstract

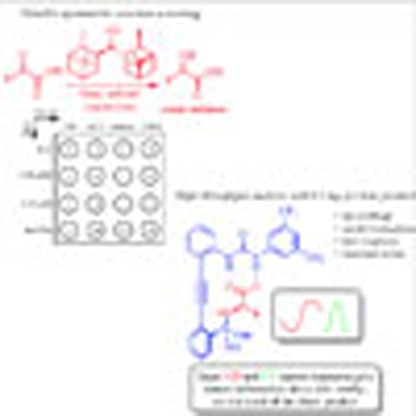

The pace of asymmetric reaction development has improved substantially with the introduction of generally available high-throughput experimentation equipment that allows parallel screening of numerous reaction parameters^1^,^2^,^3^,^4^. The overall pace of the discovery process, however, is still limited by the considerable amount of time that is required for the determination of the yield and the stereochemical outcome (enantiomeric excess and sense of asymmetric induction)—in particular when hundreds of reactions each performed on the milligram scale need to be analysed. This remaining bottleneck has pointed increasing attention to fast chromatographic methods^5^, mass spectrometry^6^,^7^,^8^, fluorescence^9^,^10^,^11^ and ultraviolet (UV)^12^,^13^ spectroscopy, infrared (IR) thermography^14^, NMR spectroscopy^15^,^16^, electrochemistry^17^, and biochemical assays^18^,^19^,^20^, all of which share the potential for high-throughput screening (HTS) of asymmetric reactions. The exceptional prospect of chiroptical sensing^21^,^22^ has encouraged the development of a variety of circular dichroism probes by Berova^23^, Anslyn^24^,^25^, Borhan^26^, Canary^27^, us^28^,^29^ and others^30^,^31^. The first steps towards real-time asymmetric reaction screening have been reported, but typically require careful handling of the sensing mixture, the use of immobilized or labelled starting materials, or product derivatization and isolation steps^32^,^33^,^34^,^35^,^36^,^37^,^38^,^39^,^40^. Despite the impressive progress in this field, new sensing strategies and probe designs that combine fast substrate binding capabilities with a distinctive chiral recognition or amplification process resulting in an immediate chiroptical sensor response suitable for quantitative ee and yield determination remain highly desirable. We now introduce a rugged sensor that achieves these goals with α-hydroxy acids even when applied to crude reaction mixtures.

Aida *et al.* have recently demonstrated the possibility of ATP binding based on cooperative salt bridge formation and covalent bonding interactions with oligomers exhibiting multiple boronic acid and guanidinium groups^41^. We reasoned that a carefully designed dual Brønsted/Lewis acid probe could exploit synergistic hydrogen bonding and dynamic covalent B–O bond formation with a chiral hydroxy acid together with substrate-to-host chirality induction to translate the molecular recognition event into a distinct chiral amplification process with a strong chiroptical signature. At the onset of this study, we anticipated that incorporation of a boronic acid and a urea moiety into a chromophoric framework that can easily accommodate a chiral bias and selectively populate a CD active conformation would generate unique opportunities for comprehensive sensing of α-hydroxy acids. The broad usefulness of the urea unit in asymmetric organocatalysis and crystal engineering applications originates from its ability to participate in directional hydrogen bonding to carboxylate and other functionalities^42^,^43^. In stark contrast to the wealth of anion sensing studies, this binding motif has barely been exploited for chirality recognition purposes and few examples of enantioselective analysis with chiral (thio)urea receptors have been reported^44^. In this regard, chiral boronic acid receptors developed by James, Anslyn and others have been more successful^45^,^46^. We therefore sought to combine the directional carboxylate binding capability of the urea motif with the compatible dynamic covalent chemistry of boronic acids into a stereodynamic diarylacetylene framework.

Here, we describe the synthesis of a novel stereodynamic Brønsted/Lewis acid receptor that forms 1:1 adducts with α- and β-hydroxy acids. The sensor design combines complementary Brønsted and Lewis acid binding sites, an approach that is conceptually related to cooperative organocatalysis^47^, with the unique stereodynamic properties of an aryl-acetylene-aryl scaffold previously used in molecular turnstiles, gyroscopes and other technomimetic devices^48^. Instantaneous analyte-to-sensor chirality imprinting generates characteristic CD and ultraviolet sensor readouts that can be correlated to the absolute configuration, enantiomeric composition and total amount of the target compounds. The general utility and ruggedness of this approach is demonstrated by analysis of the yield, ee and sense of asymmetric induction of crude reaction mixtures obtained by Ipc_2_BCl reductions of an α-keto acid.

## Results

### Sensor design and preparation

**Figure 1:**
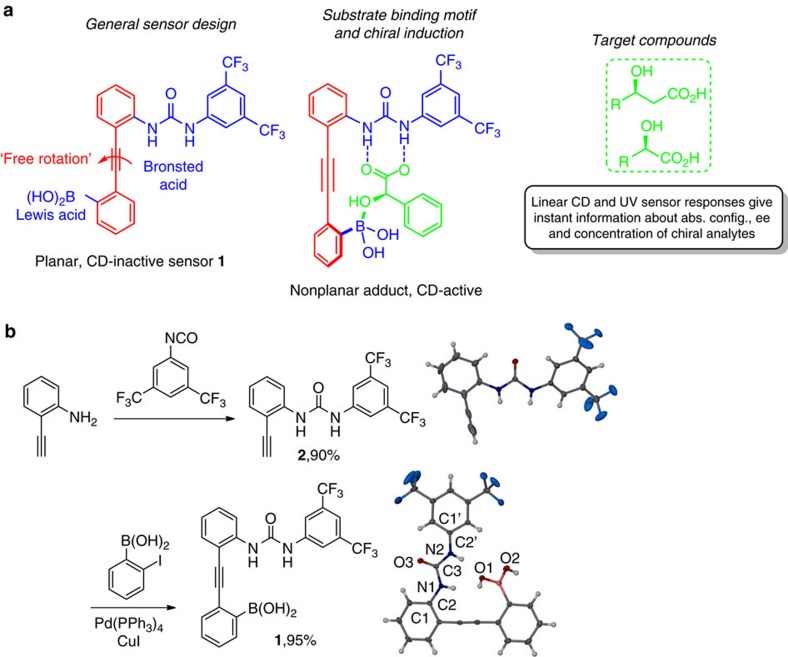
Design and synthesis of the stereodynamic sensor 1. (**a**) Synergistic substrate binding with complementary Brønsted/Lewis acid sites and chiral amplification. (**b**) Synthesis of **1** and crystal structures of **1** and **2**. Selected parameters for **1**: Bond length [Å]: CH1- - -O3: 2.292, CH1′- - -O3: 2.235, NH1- - -O1: 2.288 and NH2–O1: 2.005. Bond angles [deg]: C1–C2–N1–C3: 6.6 and C1′–C2′–N2–C3: 2.7.

The Brønsted/Lewis acid probe **1** displays several carefully selected features (Fig. 1a). First, fixation of hydroxy acids occurs at two spatially separated sites and involves directional hydrogen bonding between the carboxylate group and the urea unit and reversible formation of a dative B–O bond of the alcohol group to the boronic acid moiety^49^. Second, the central triple bond affords a rigid host structure that maintains—in its free form—intrinsic rotational freedom about the elongated axis which is a prerequisite for spontaneous chiral amplification. Third, **1** consists of a fully conjugated π-system to shift the chiroptical sensor response above 300 nm, that is, to higher wavelengths than the individual phenylboronic acid and diphenylurea moieties. This is important as it reduces possible interference from impurities with intrinsic CD signals below 300 nm that could be present in crude reaction mixtures; for example, chiral catalysts, reagents or by-products^21^,^22^,^50^. Fourth, the complementary nature and location of the two binding units in **1** favour formation of a highly organized host–guest adduct. This sets the stage for effective imprinting of the substrate chirality onto the aryl-acetylene-aryl axis in **1** under reversible conditions and thermodynamic control. This asymmetric transformation of the first kind is controlled by three forces: the two binding motifs and the minimization of steric repulsion.

**Figure 2:**
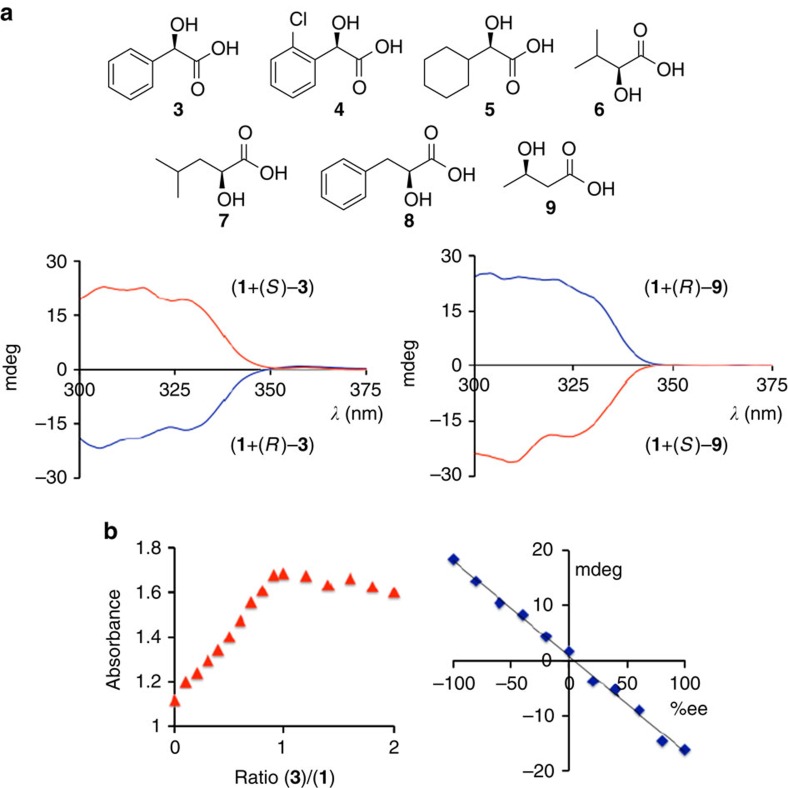
Chiroptical sensing of hydroxy acids. (**a**) Structures of hydroxy acids investigated and CD sensing of the enantiomers of **3** and **9**. The CD measurements were conducted immediately after mixing the substrate, Et_3_N and **1** at 1.80 × 10^−4^ M in acetonitrile. The CD effects induced by (*R*)-**3** and (*R*)-**9** are shown in blue and the CD responses to the (*S*)-enantiomers of **3** and **9** are shown in red. (**b**) Ultraviolet absorbance change of **1** upon addition of **3** (left) and linear CD response of **1** to the enantiomeric composition of **3** (right). All measurements were conducted immediately after mixing the substrate, Et_3_N and **1** at 1.80 × 10^−4^ M in ACN.

We started our investigation with a two-step synthesis of **1** that avoids functional group protection steps. The urea compound **2** was prepared in 90% yield from 2-aminophenylacetylene and 3,5-bis(trifluoromethyl)phenylisocyanate (Fig. 1b). The target compound was then directly assembled by Sonogashira coupling of **2** with 2-iodophenylboronic acid at room temperature. We obtained **1** in almost quantitative yields and did not observe any sign of the formation of Suzuki coupling by-products. Crystallographic analysis of **1** showed a planar molecular arrangement which is stabilized by two C_aryl_H- - -O=C bonds within the 1,3-diarylurea moiety and intramolecular hydrogen bonding between the urea unit and the boronic acid. With the probe in hand, we set out to test the usefulness of **1** for chirality sensing using a series of hydroxy acids. While **1** is CD-silent in the absence of a chiral substrate, it gave strong induced CD (ICD) signals above 300 nm and at low concentrations upon addition of the enantiomers of hydroxy acids **3**–**9** (Fig. 2a). With all (*S*)-2-hydroxy acids tested we found that **1** generates a positive ICD signal and we observed a negative chiroptical response when the (*R*)-enantiomers were used (see Supplementary Figs 1–8). The chiroptical readout of **1** in the presence of the 3-hydroxy acid **9** was equally distinct albeit with the opposite ICD sense. Importantly, the substrate binding and the appearance of the ICD response of **1** occur instantaneously. With a simple mix-and-measure procedure one can thus rapidly determine the absolute configuration of minute amounts of α- and β-hydroxy acids.

### Sensing studies

The favourable chiroptical properties of **1** encouraged us to explore the possibility of quantitative concentration and ee analysis. We were pleased to find that the host–guest interactions result in a steady increase in the ultraviolet absorbance of **1** until more than one equivalent of the hydroxy acid is added (Fig. 2b, Supplementary Figs 12–16). Unlike the ICD effect discussed above, this optical response is not enantioselective and therefore provides an orthogonal means to determine the total amount or concentration of the substrate regardless of the sample ee. When we analysed the ICD amplitudes of **1** in the presence of nonracemic **3,** we obtained a perfectly linear relationship between the CD signals at 300 and 330 nm and the enantiomeric composition of the substrate (Fig. 2b, Supplementary Figs 9–11). Altogether, the substrate binding event and the concomitant reorganization of **1** yield spontaneous ultraviolet and CD sensor responses that can be correlated to the chirality (absolute configuration and ee) and the total amount of the guest. Using the previously quantified chiroptical readouts of **1,** we were able to determine the concentration and enantiomeric composition of 10 samples with good accuracy (Table 1). The values obtained by chiroptical chemosensing with **1** were generally within a few per cent of the actual concentration and ee. The nexus of instantaneous dual-site binding of hydroxy acid targets, strong chiral amplification and quantifiable ultraviolet/CD sensing responses at low concentration implied to us that the chemsosensor **1** could be used for fast determination of the absolute configuration, yield and ee of submillimolar sample amounts even in the presence of other compounds. Such a rugged method would be applicable to direct HTS of crude asymmetric reaction mixtures that typically contain remaining starting materials, additives, reagents, by-products etc.

### Mechanistic studies

**Figure 3:**
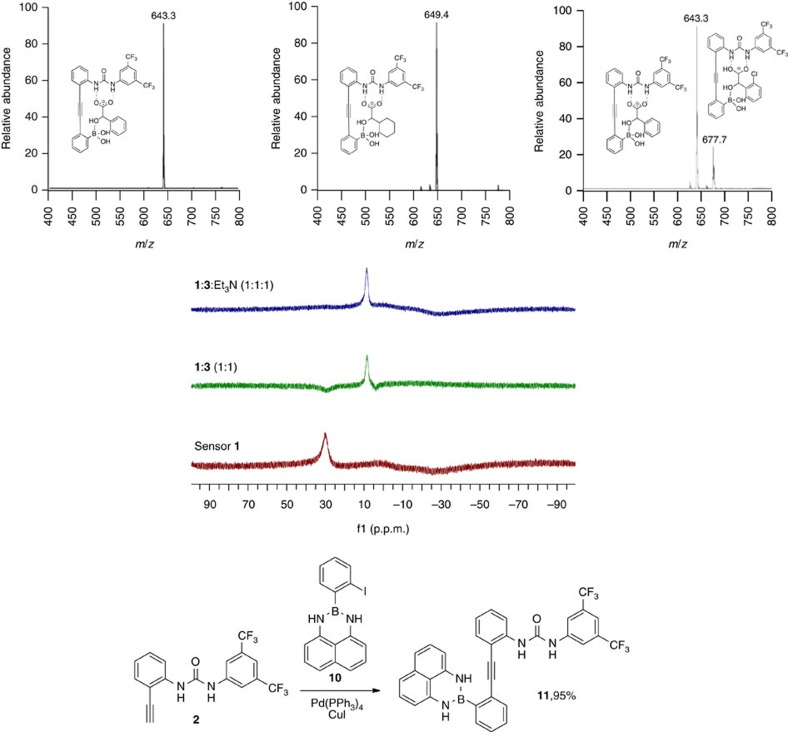
Binding studies. Top: ESI/MS detection of the adducts formed from **1** and mandelic acid **3** (left) and hexahydromandelic acid **5** (middle) in the presence of one equivalent of Et_3_N. The formation of **1·**(*R*)-**4** upon addition of (*R*)-**4** to **1·**(*R*)-**3** in acetonitrile is shown on the right. Middle: ^11^B NMR analysis of **1** (red), **1** and **3** (green), and **1** and **3** in the presence of Et_3_N (blue) in ACN-d_3_. Bottom: Synthesis of the Dan-protected analogue **11**.

The synergistic substrate binding motif involving hydrogen bonding between the carboxylate and the urea unit, and formation of a covalent bond between the hydroxyl group and the boronic acid moiety depicted in Fig. 1, was verified by electrospray ionization-mass spectrometry (ESI-MS) and NMR analysis. Electrospray mass spectrometry (negative ion mode) of an acetonitrile solution containing **1**, a hydroxy acid and stoichiometric amounts of triethylamine yielded strong signals for the 1:1 adducts **1·3** and **1·5** (Fig. 3). A titration experiment with 2-chloromandelic acid, **4**, confirmed that the substrate binding is reversible which is consistent with dynamic B–O bond formation. Addition of (*R*)-**4** to a solution containing preformed adduct **1·**(*R*)-**3** in acetonitrile showed partial conversion to **1·**(*R*)-**4** according to ESI-MS analysis. It is noteworthy that the MS analyses were performed under the same conditions as the chiroptical measurements and other species were not detected. Addition of 2 equivalents of **3** to sensor **1** did not show any sign of formation of 2:1 or higher order aggregates by MS and NMR analysis (Supplementary Figs 17 and 20). The anticipated hydrogen bonding interactions were evident from proton NMR titration experiments showing strong downfield shifts of the urea protons in **1** upon addition of the carboxylate of **3** (Supplementary Fig. 20). The B–O bond formation was investigated by ^11^B NMR spectroscopy using **1** and **3** as well as *O*-acetyl mandelic acid. It is known that sp^2^-hybridized boron compounds including free boronic acids show ^11^B NMR signals at ∼30 p.p.m., while sp^3^ hybridization typically results in characteristic upfield shifts and signals between 0 and 10 p.p.m.^51^. The ^11^B NMR signal of the free boronic acid unit in **1** showed a signal around 30 p.p.m. which was shifted below 10 p.p.m. when one equivalent of mandelic acid, **3**, was added (Fig. 3). Essentially the same results were obtained in the presence and absence of triethylamine. While this confirms formation of the B–O bond with **3**, no ^11^B shift was observed with *O*-acetyl mandelic acid as expected (Supplementary Figs 21 and 22). The sensitivity of the substrate binding and chirality imprinting process to the presence of water was tested. Titration of up to 10 equivalents of water into an acetonitrile solution containing **1**, Et_3_N and substrate **3** did not reduce the chiroptical sensor response (Supplementary Fig. 23). The absence of interference of small amounts of water is important and underscores the ruggedness of the sensing assay.

Finally, we prepared the 1,8-diaminonaphthalene (Dan) protected analogue **11** to probe the importance of the covalent B–O bond for the chiral induction and chiroptical sensing mechanism. The attachment of the Dan group to the boronic acid function has been used to mask its reactivity in Suzuki cross-coupling reactions, presumably by interfering with the formation of an activated tetrahedral intermediate^52^. While **3** was expected to undergo hydrogen bonding to the urea unit in **11**, the lack of complementary B–O bond formation—the B(Dan) moiety cannot accommodate the hydroxyl group of **3**—should largely diminish imprinting of the substrate chirality on the stereodynamic sensor scaffold. Indeed, we did not observe an ICD signal using **3**, **11** and Et_3_N in acetonitrile under the conditions described in Fig. 2. This proves that the dual-site binding motif is a prerequisite for effective chirality sensing. The significance of steric interactions in the chiral amplification process and chiroptical sensor readout is evident from a comparison of the CD effects observed with the aliphatic α-hydroxy acids **5**–**7**. Sensor **1** shows a stronger CD response to **5** and **6** carrying a branched cyclohexyl or isopropyl residue attached to the chiral centre compared with **7** which has a remotely branched isobutyl group (see Supplementary Figs 4–6).

### Asymmetric reaction screening

**Figure 4:**
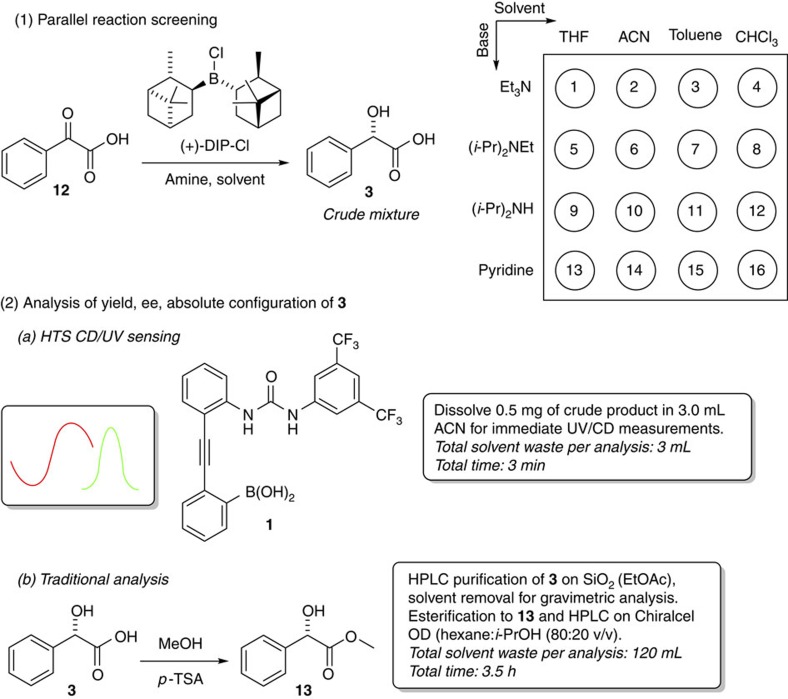
Reaction analysis. Top: Set-up of 16 variations of the reduction of phenylglyoxylic acid, **12**, with (+)-Ipc_2_BCl (combinations of 4 solvents and 4 bases). Bottom: Comparison of chiral chemosensing and traditional reaction analysis.

**Figure 5:**
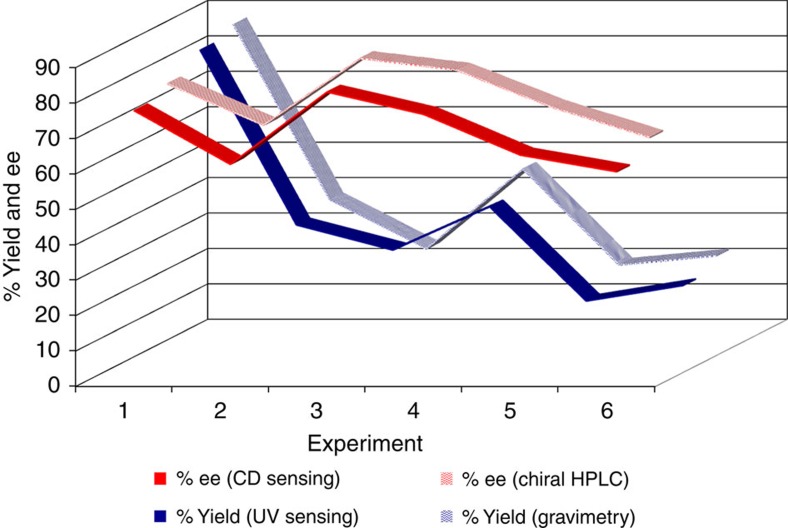
Comparison with existing methods. Comparison of the ee's (red) and yields (blue) obtained by the traditional approach and chiroptical sensing with 1.

Having established the substrate scope, the chiral recognition mechanism and the practicality of chiroptical sensing with the stereodynamic Brønsted/Lewis acid probe **1**, we decided to evaluate the possibility of HTS of an asymmetric reaction. For this purpose, we chose the reduction of phenylglyoxylic acid, **12**, with (+)-B-chlorodiisopinocampheylborane, (+)-Ipc_2_BCl, and we set-up a total of 16 reactions with varying solvents and amine additives (Fig. 4, Supplementary Figs 24 and 25)^53^. Each reaction mixture was charged with **12** (10.0 mg), slight access of (+)-Ipc_2_BCl and stirred under air for 12 h at room temperature. Upon quenching of the reaction and solvent removal, 0.5 mg of the *crude* residue was removed for direct ultraviolet and CD measurements, and the remaining material was subjected to traditional gravimetric analysis after isolation of **3** by flash chromatography and chiral high-performance liquid chromatography (HPLC) separation of the methyl ester **13**. For chiroptical sensing, the crude residue, **1** and triethylamine were dissolved in 3 ml of acetonitrile and the samples were analysed without delay. Because the CD sensing of the product mixtures from experiments 6 to 9 and 11 to 16 did not show a measurable ee, the outcome of these reactions was not further investigated. All other reactions were fully examined. Two simple ultraviolet and CD measurements using the mix-and-measure protocol with sensor **1** allowed fast determination of the yield, ee and the absolute configuration of the reduction product (Table 2 and Figure 5). It is noteworthy that ultraviolet and CD spectra are typically obtained together with common CD spectrometers which greatly simplifies the sensing protocol. The comparison of the results from the chirality sensing method and the traditional reaction analysis reveals that the yields and ee's are close even though one could expect some interference between the sensor and the boron reagent or derivatives thereof that might be still present in the crude mixture. Error margins within a few per cent, however, are acceptable for HTS efforts which generally have the objective to rapidly identify reaction conditions that afford the most superior results. In all cases, the sensor reported the correct absolute configuration of the major enantiomer formed. Yields and ee's of **3** were successfully quantified irrespective of the reaction conversion (compare entries 1 and 3, for example). The quantitative ultraviolet and CD analyses were performed using the previously established chiroptical probe readouts, and recalibration experiments were not necessary which greatly enhances the efficiency and practicality of the crude reaction analysis. Altogether, the chiroptical sensing method required 3 ml of solvent per sample and was completed within 3 min. By contrast, the isolation of **3** by flash chromatography for gravimetric yield determination and the esterification toward **13** for subsequent chiral HPLC analysis required 120 ml and 3.5 h per sample. The successful HTS of the asymmetric Ipc_2_BCl reduction of the α-keto acid 12 underscores the ruggedness and usefulness of chirality chemosensing with 1, and it coincides with a dramatic decrease in labour, analysis time and waste production.

In conclusion, the dual-site binding of α- and β-hydroxy acids by the Brønsted/Lewis acidic probe **1** and concomitant chirality imprinting onto the stereodynamic sensor scaffold generate characteristic chiroptical readouts that can be correlated to the absolute configuration, enantiomeric composition and total amount of the target compounds. A series of ultraviolet, CD, NMR and MS experiments confirmed the host–guest interactions and the operative chirality recognition and reporting modes. The HTS utility was demonstrated by comprehensive analysis (yield, ee and sense of asymmetric induction) with just 0.5 mg of crude reaction mixtures obtained by Ipc_2_BCl reductions of an α-keto acid. This approach eliminates the general need for reaction work-up, product isolation and recalibration of spectroscopic readouts, and it reduces analysis time, labour and waste production. Altogether, this study shows how chiroptical sensing accelerates asymmetric reaction development efforts on the milligram scale while increasingly demanding cost accounting and sustainability standards are addressed.

## Methods

### Asymmetric reaction screening

Solutions of phenylglyoxylic acid, **12** (10 mg, 0.06 mmol), an amine additive (0.06 mmol) and (+)-DIP-Cl (21.4 mg, 0.067 mmol) in 0.5 ml of anhydrous solvent were stirred in 4 ml vials under air for 12 h at room temperature. The reaction was quenched with 1 M NaOH (100 μl, 0.1 mmol) and H_2_O_2_ (10 μl, 30% in H_2_O, 0.3 mmol) and stirred for 30 min. Then, 1 M HCl was added (150 μl) and the solvent was removed *in vacuo*. From the crude reaction mixture, 0.5 mg of the white solid were used for ultraviolet and CD analysis. For traditional analysis (gravimetry and chiral HPLC), the remaining portion of the material was purified by flash chromatography on silica gel (EtOAc) to give **3** as a white solid. Each column consumed ∼100 ml of solvent and required ∼16 min, including column packing, collection and solvent removal. For chiral HPLC analysis, **3** was converted to the methyl ester **13** by refluxing in 5 ml of anhydrous methanol for 3 h in the presence of *p*-TSA (0.1 molar equivalent). The ee of the methyl mandelate **13** was determined by HPLC on a Chiralcel OD column using hexane:*i*-PrOH (80:20 v/v) as mobile phase at 1 ml min^−1^, *t*_1_, (*R*)=5.6 min, *t*_2_, (*S*)=9.2 min and required ∼15 ml of solvent and ∼12 min per sample, see Supplementary Figs 26–31. The substrate binding and sensing mechanism were investigated by NMR, CD, and MS, see Supplementary Figs 17–23. For more information on the quantitative chirality analysis and crystallography, see Supplementary Methods, Supplementary Figs 35 and 36 and Supplementary Tables 1–6.

### Synthetic protocols

All reagents and solvents were commercially available and used without further purification. Reactions were carried out under inert atmosphere and anhydrous conditions unless stated otherwise. Flash chromatography was performed on silica gel, particle size 40–63 μm. NMR spectra were obtained at 400 MHz (^1^H NMR) and 100 MHz (^13^C NMR) using ACN-d_3_ as solvent, see Supplementary Figs 32–34.

### *N*-(2-ethynylphenyl)-*N′*-(3,5-bis(trifluoromethyl)phenyl)urea, **Compound 2**

A solution of 2-ethynylaniline (200 mg, 1.71 mmol) and 3,5-bis(trifluoromethyl)phenyl isocyanate (521.1 mg, 2.05 mmol) was stirred in 8 ml of anhydrous CH_2_Cl_2_ for 12 h. The precipitate was filtered and washed with cold CH_2_Cl_2_ to afford 573.3 mg (1.54 mmol, 90%) of a white solid. ^1^H NMR *δ*=3.88 (s, 1H), 7.04 (dd, *J*=7.6 Hz, 7.6 Hz, 1H), 7.38 (dd, *J*=8.7 Hz, 7.6 Hz, 1H), 7.47 (d, *J*=8.7 Hz, 1H), 7.60–7.61 (m, 2H), 8.05 (s, 2H), 8.20 (d, *J*=7.6 Hz, 1H), 8.38 (s, 1H). ^13^C NMR *δ*=81.5, 88.0, 113.7, 120.0, 121.1, 126.2 (q, *J*=271 Hz), 125.4, 128.0, 133.0 (q, *J*=67 Hz), 134.4, 135.1, 143.1, 144.0, 154.6. Anal. calcd. for C_17_H_10_F_6_N_2_O: C, 54.85; H, 2.71; N, 7.53. Found: C, 55.04; H, 2.65; N, 7.54.

### 2-[2′-*N*-(3,5-bis(trifluoromethyl)phenylureido))phenylethynyl]phenylboronic acid, **compound 1**

A solution of **2** (200 mg, 0.54 mmol), 2-iodophenylboronic acid (200 mg, 0.81 mmol), Pd(PPh_3_)_4_ (31.2 mg, 0.027 mmol), CuI (5.1 mg, 0.027 mmol) and ethanolamine (130 μl, 2.16 mmol) was stirred at room temperature in 10 ml of anhydrous tetrahydrofuran for 12 h. The mixture was quenched with water and extracted with CH_2_Cl_2_. The combined organic layers were dried over MgSO_4_ and concentrated *in vacuo*. Purification by flash chromatography on silica gel (hexanes:EtOAc 4:1) afforded 252.5 mg (0.51 mmol, 95%) of a yellow solid. ^1^H NMR *δ*=6.84 (s, 2H), 7.10 (dd, *J*=8.6 Hz, 7.5 Hz, 1H), 7.39–7.55 (m, 4H), 7.64–7.66 (m, 2H), 7.74 (d, *J*=8.0 Hz, 1H), 8.07 (s, 1H), 8.14 (s, 2H), 8.29 (d, *J*=8.6 Hz, 1H), 8.60 (s, 1H). ^13^C NMR *δ*=89.4, 91.9, 109.9, 114.4, 118.0, 118.1, 118.1, 121.0, 121.1, 121.5, 125.4, 130.9, 132.7 (q, *J*=264 HZ), 134.0, 134.5 (q, *J*=26 Hz), 136.2, 142.7, 144.1, 149.6, 154.7. Anal. calcd. for C_23_H_15_BF_6_N_2_O_3_: C, 56.13; H, 3.07; N, 5.69. Found: C, 55.85; H, 2.93; N, 5.52.

### Data availability

All relevant data are available from the authors upon request. Crystallographic data are available free of charge from The Cambridge Crystallographic Data Centre via www. ccdc.cam.ac.uk/data_request/cif. The CCDC numbers are 1452256 for **1** and 1452255 for **2**.

## Additional information

**How to cite this article:** Bentley, K. W. *et al.* Chirality imprinting and direct asymmetric reaction screening using a stereodynamic Brønsted/Lewis acid receptor. *Nat. Commun.* 7:12539 doi: 10.1038/ncomms12539 (2016).

## Supplementary Material

Supplementary Information Supplementary Figures 1-36, Supplementary Tables 1-6 and Supplementary Methods

## Figures and Tables

**Table 1:** Quantitative sensing of nonracemic samples of **3**.

Sample	Conc. (mM)	Ultraviolet* sensing	Deviation (%)	Sample	Actual %ee (*R*)	CD† sensing	Deviation (%)
1	0.56	0.59	5.4	6	87.0	88.5	1.7
2	1.01	1.07	5.9	7	76.0	75.1	1.2
3	2.36	2.38	0.8	8	12.0	12.3	2.5
4	2.93	2.95	0.7	9	−26.0	−26.8	3.1
5	3.34	3.37	0.9	10	−68.0	−68.8	1.2

^*^Averaged value from the ultraviolet responses at 320 and 330 nm.

^†^Averaged value from the CD responses at 300 and 330 nm.

**Table 2:** Results of the reaction analysis.

(+)-Ipc_2_BCl reduction			Traditional analysis		Chiroptical sensing	
Run	Base	Solvent	Yield (%)	ee (%) and abs. config.	Yield (%)	ee (%) and abs. config.
1	Et_3_N	THF	86.6	79.3 (*S*)	84.8	77.1 (*S*)
2	Et_3_N	ACN	37.1	67.6 (*S*)	34.3	61.1 (*S*)
3	Et_3_N	Toluene	23.2	86.4 (*S*)	25.0	80.9 (*S*)
4	Et_3_N	CHCl_3_	45.7	83.1 (*S*)	40.2	76.6 (*S*)
5	(*i*-Pr)_2_NEt	THF	18.6	72.5 (*S*)	14.0	64.4 (*S*)
10	(*i*-Pr)_2_NH	ACN	21.1	64.1 (*S*)	17.8	58.7 (*S*)

ACN, acetonitrile; THF, tetrahydrofuran.
