# Supplementary material for: Chirality imprinting and direct asymmetric reaction screening using a stereodynamic Brønsted/Lewis acid receptor
Source: Nat Commun. 2016 Aug 23;7:12539. doi: 10.1038/ncomms12539 (PMC4996974; doi:10.1038/ncomms12539)
Supplement: Supplementary Information — Supplementary Figures 1-36, Supplementary Tables 1-6 and Supplementary Methods [file ncomms12539-s1.pdf]

Supplementary Figure 1. Structures of substrates tested with **1**. Only one enantiomer is shown.

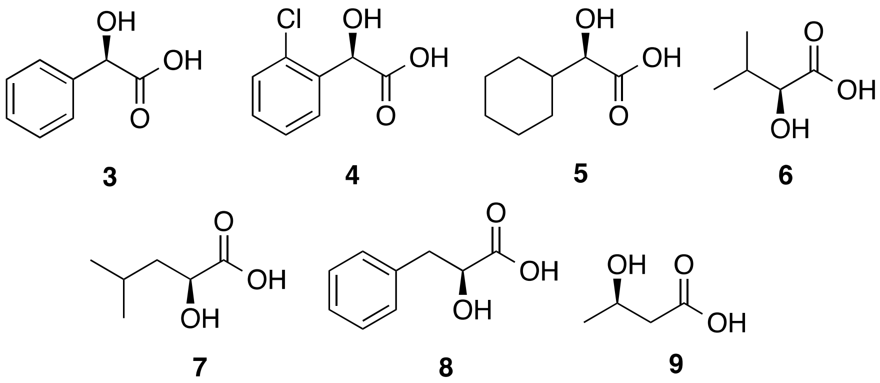

Supplementary Figure 2. CD spectra obtained using **1** and (*R*)-**3** (blue) and (*S*)-**3** (red)

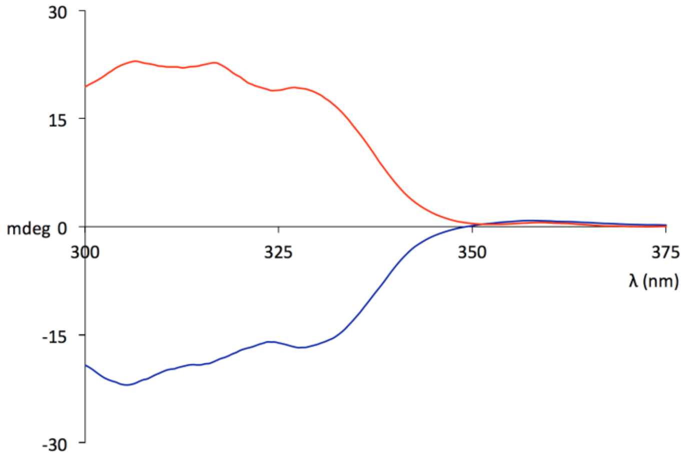

Supplementary Figure 3. CD spectra obtained using **1** and (*R*)-**4** (blue) and (*S*)-**4** (red)

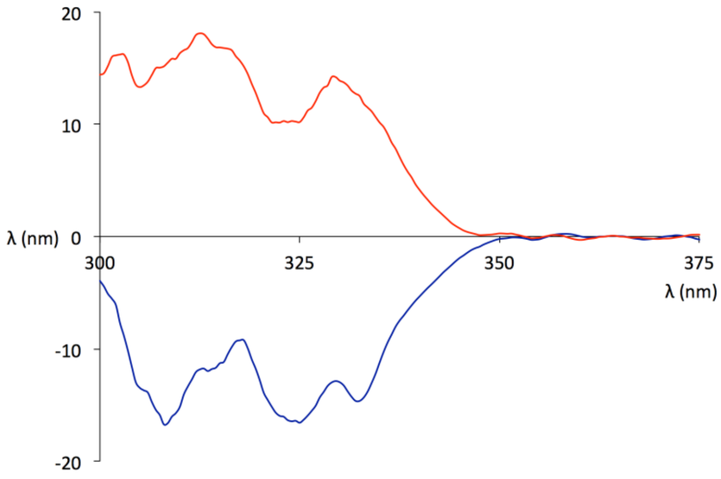

Supplementary Figure 4. CD spectra obtained using **1** and (*R*)-**5** (blue) and (*S*)-**5** (red)

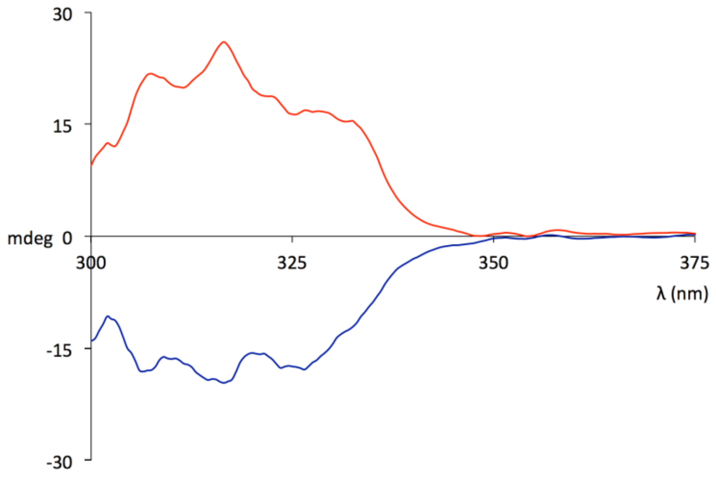

Supplementary Figure 5. CD spectra obtained using **1** and (*R*)-**6** (blue) and (*S*)-**6** (red)

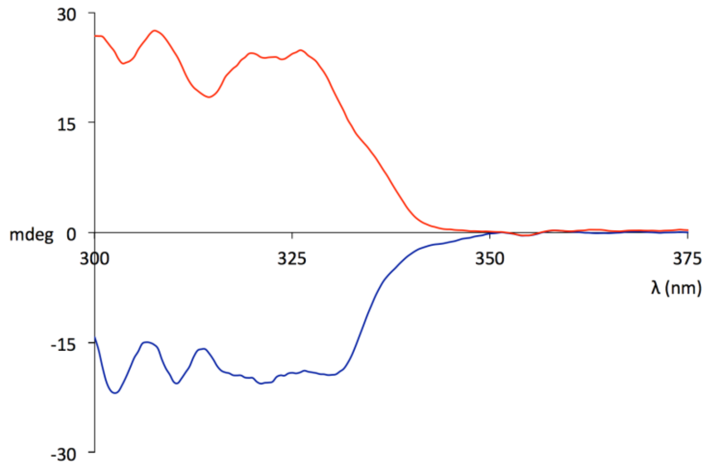

Supplementary Figure 6. CD spectra obtained using **1** and (*R*)-**7** (blue) and (*S*)-**7** (red)

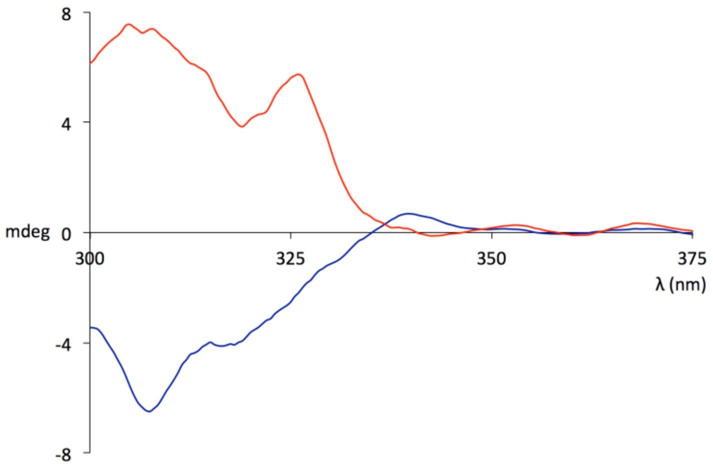

Supplementary Figure 7. CD spectra obtained using **1** and (*R*)-**8** (blue) and (*S*)-**8** (red)

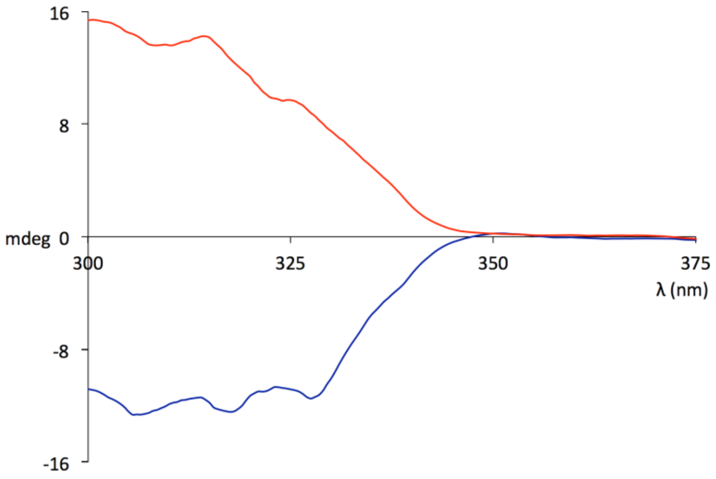

Supplementary Figure 8. CD spectra obtained using **1** and (*R*)-**9** (blue) and (*S*)-**9** (red)

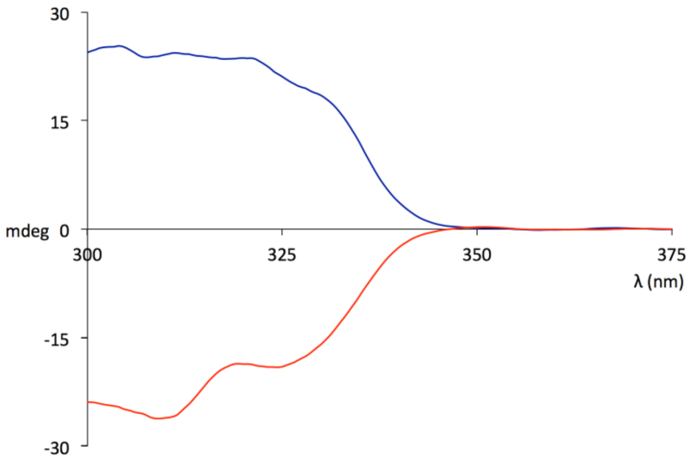

Supplementary Figure 9. CD Spectra of the complex obtained with **1** and scalemic samples of **3**

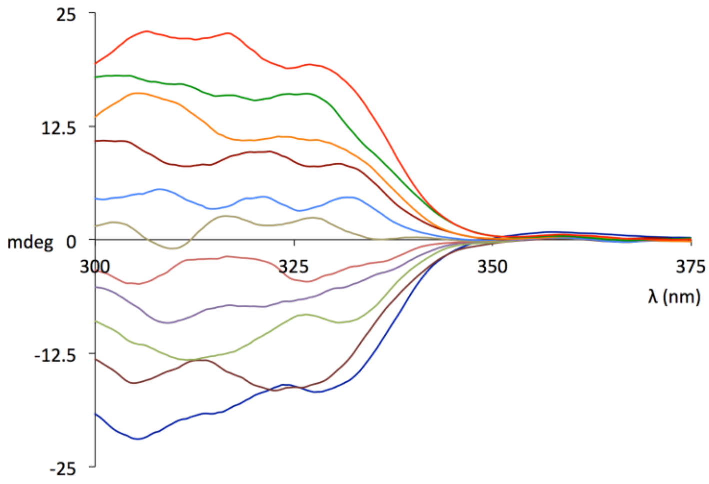

Supplementary Figure 10. Linear relationship between the CD amplitudes at 300 nm and the enantiomeric excess of **3**

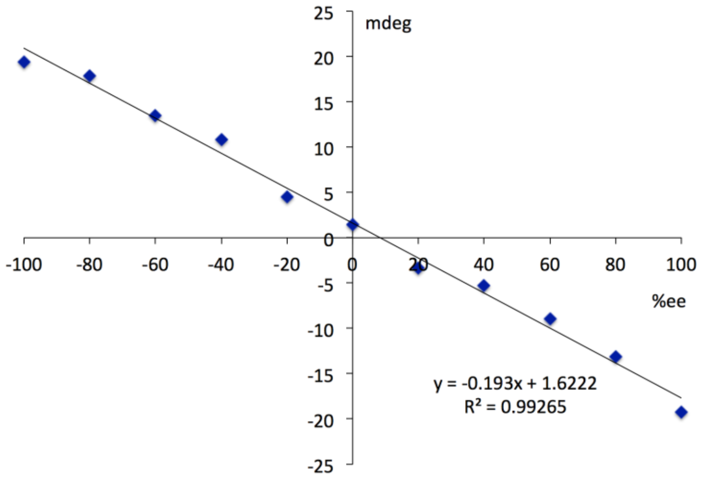

Supplementary Figure 11. Linear relationship between the CD amplitudes at 330 nm and the enantiomeric excess of **3**

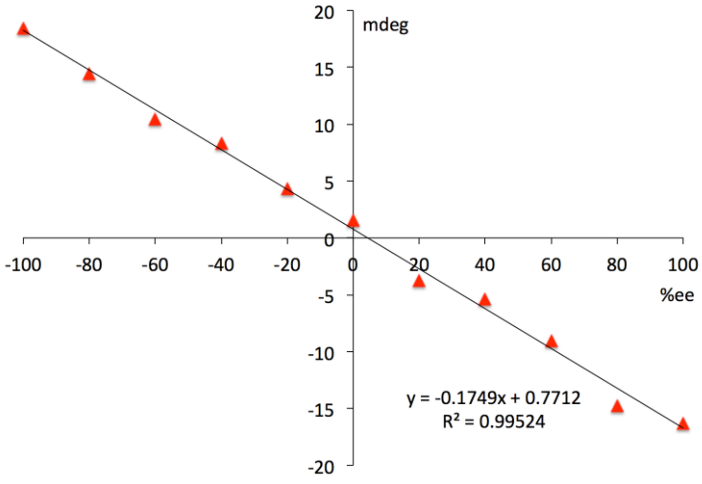

Supplementary Figure 12. UV Spectra of **1** upon addition of **3** in varying molar ratios from 0 to 100 mol% (blue) and from 120 to 200 mol% (red)

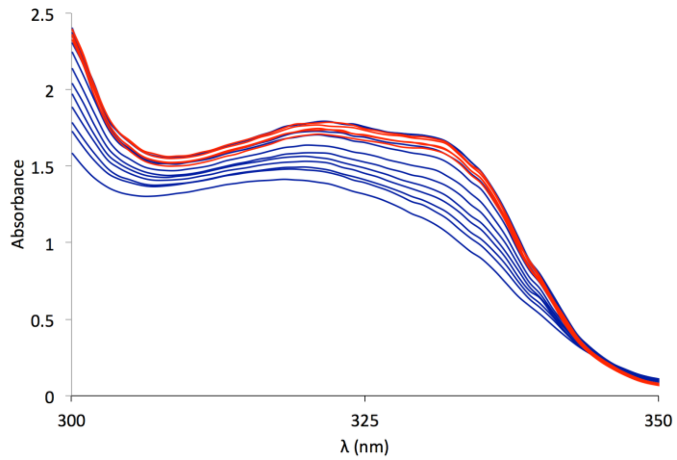

Supplementary Figure 13. Plot of the absorbance of **1** at 320 nm from 0 to 200 mol% of **3**

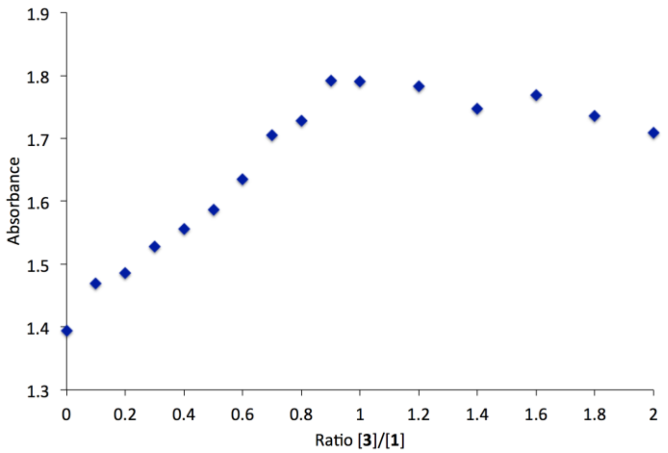

Supplementary Figure 14. Curve fitting of the absorbance of **1** at 320 nm from 0 to 100 mol% of **3**

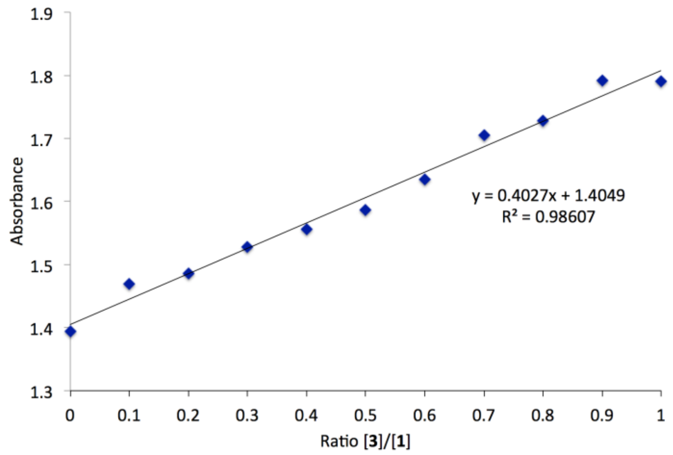

Supplementary Figure 15. Plot of the absorbance of **1** at 330 nm from 0 to 200 mol% of **3**

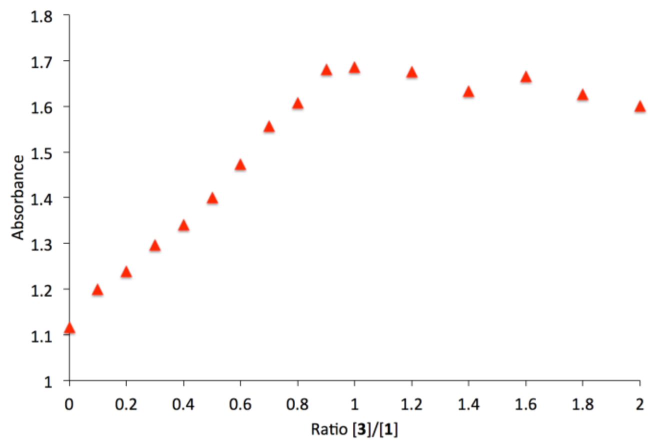

Supplementary Figure 16. Curve fitting of the absorbance of **1** at 330 nm from 0 to 100 mol% of **3**

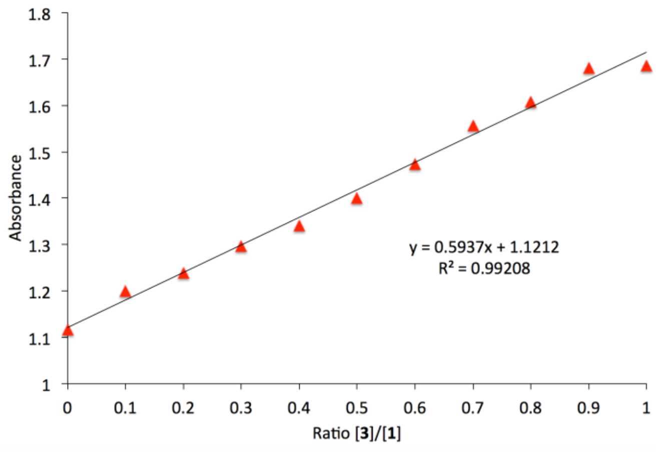

Supplementary Figure 17. MS Spectrum of the complex obtained with **1** and **3**

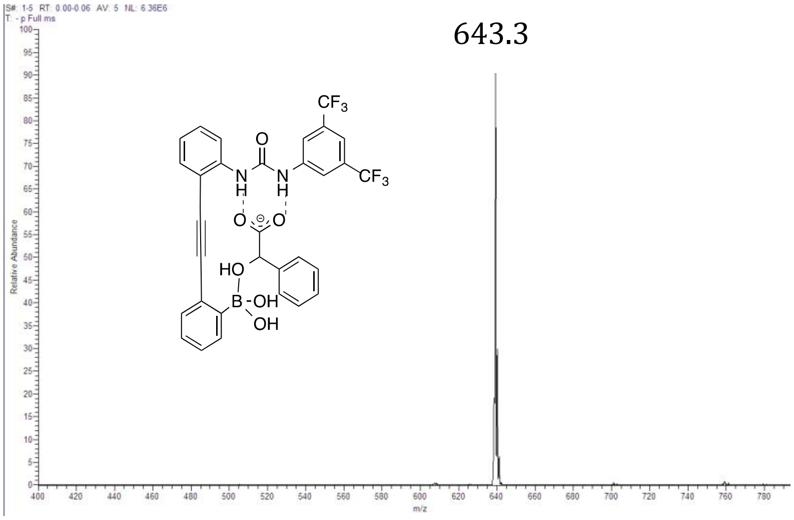

ESI-MS: m/z = 643.3 (M$^-$)

Supplementary Figure 18. MS Spectrum of the complex obtained with **1** and **5**

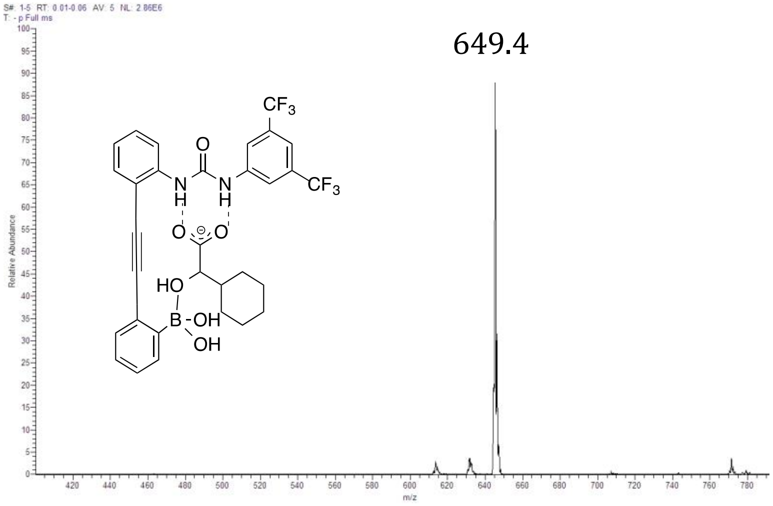

ESI-MS: m/z = 649.4 (M$^-$)

Supplementary Figure 19. MS Titration experiment

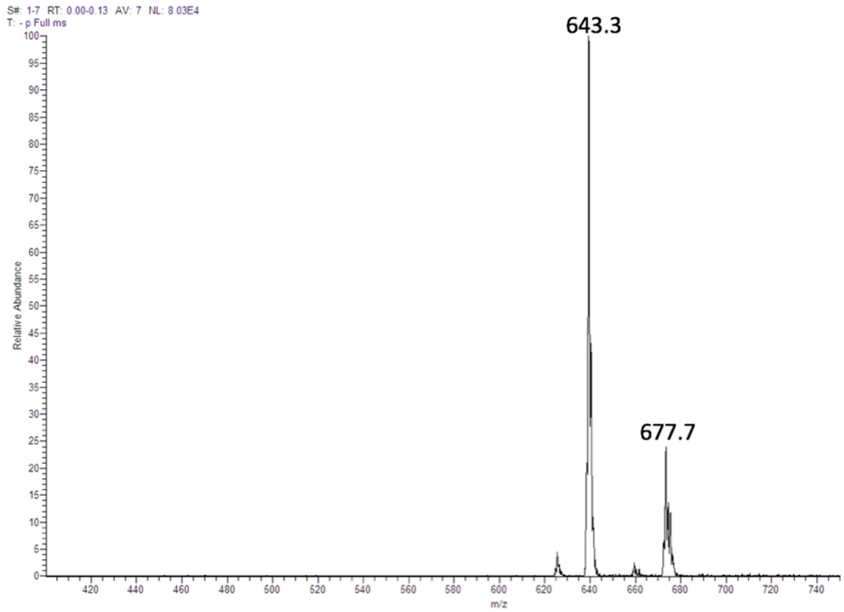

ESI-MS: m/z = 643.3 (M^-), 677.7 (M^-)

Supplementary Figure 20. ^{1}H NMR spectra of **1** (10 mg, 0.02 mmol) with **3** and Et_3N at various amounts (0.0, 0.2, 0.4, 0.6, 0.8, 1.0, 1.2 and 2.0 equivalents) were collected. Instant downfield shifts of the urea protons were observed as the amount of **3** was varied from 0.0 equivalents (red) to 2.0 equivalents (violet).

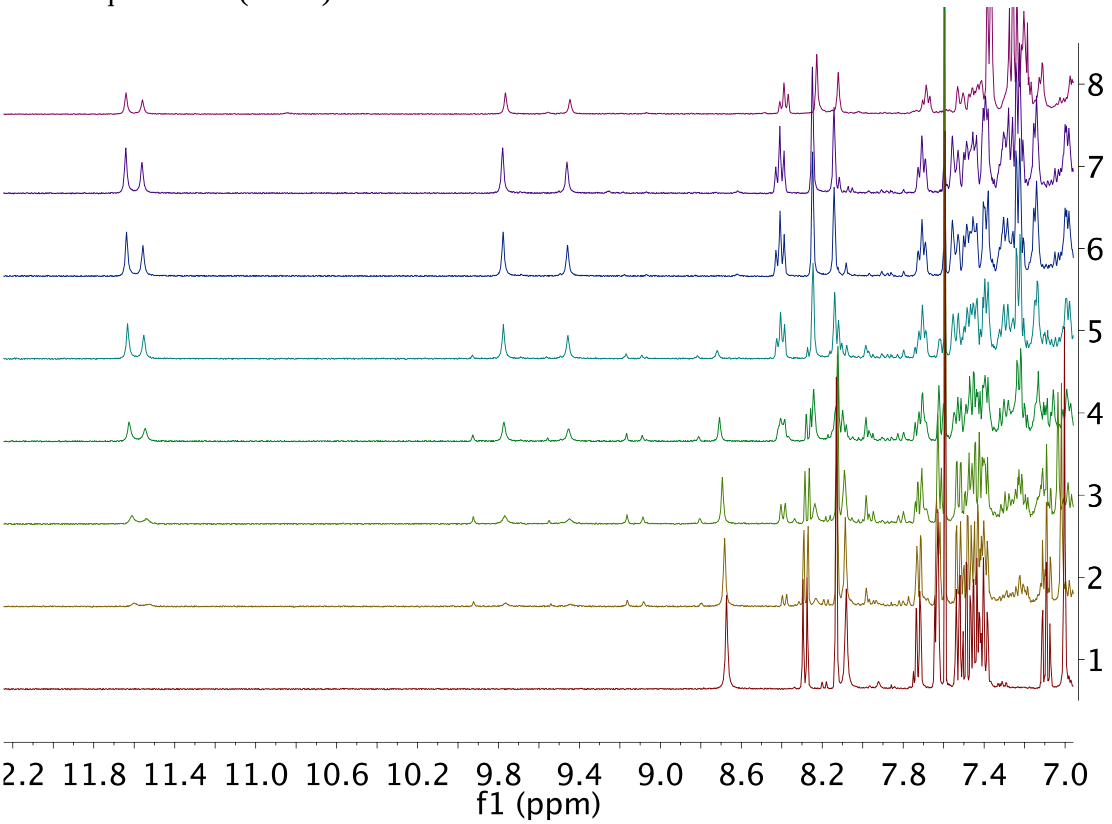

Supplementary Figure 21. ^{11}B NMR spectra of **1** (red), **1** and **3** (green), and **1**, **3**, and Et_3N (blue).

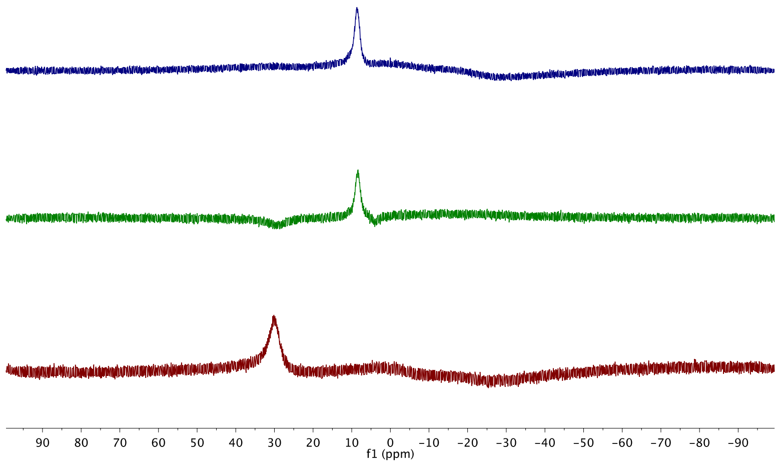

Supplementary Figure 22. ^{11}B NMR spectra of **1** (red) and **1** and with Et_3N and **14** (green).

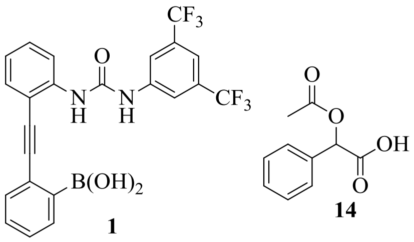

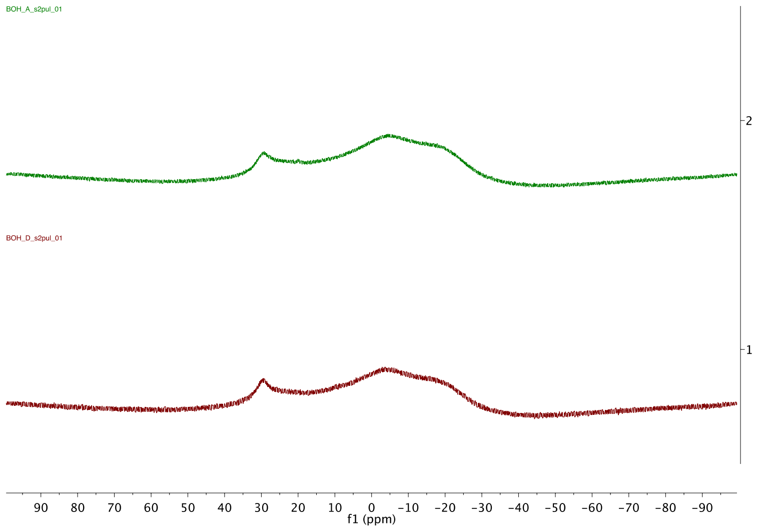

Supplementary Figure 23. Water sensitivity testing.

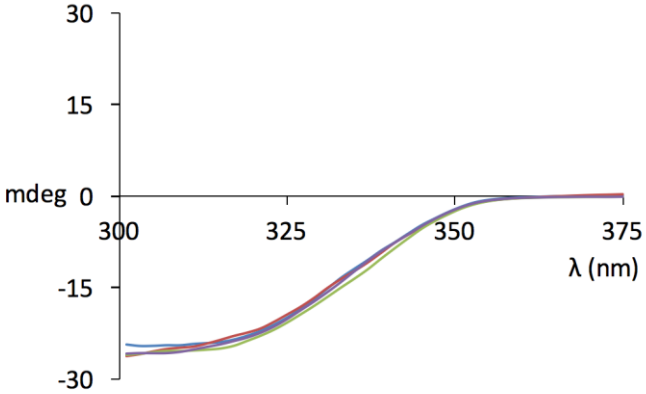

Supplementary Figure 24. Asymmetric reduction of **12**.

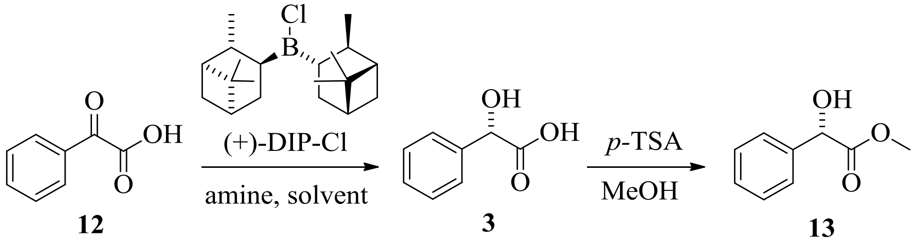

Supplementary Figure 25. Reaction screening setup:

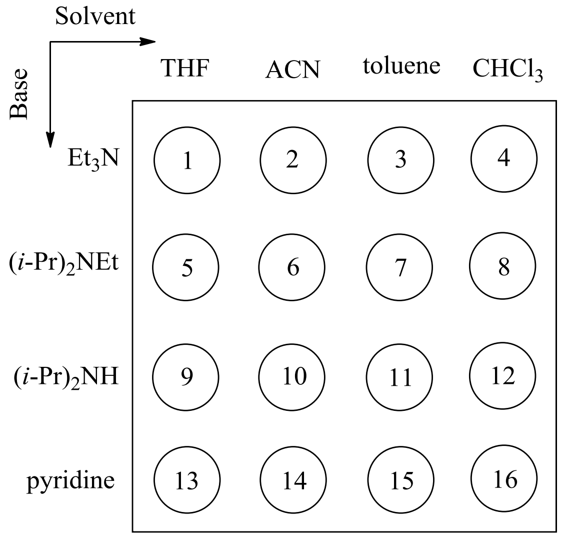

Supplementary Figure 26. HPLC separation of the esterification product from experiment 1

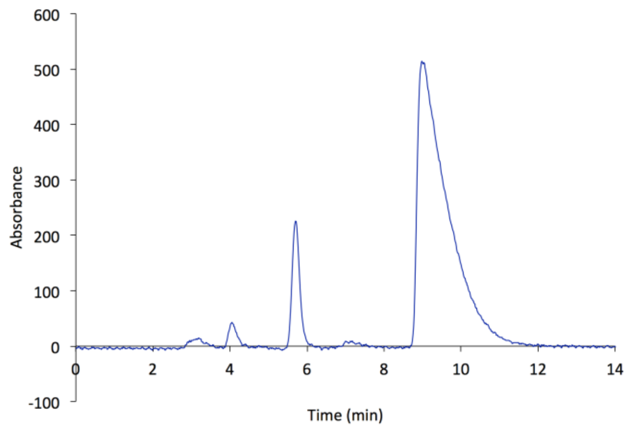

Supplementary Figure 27. HPLC separation of the esterification product from experiment 2

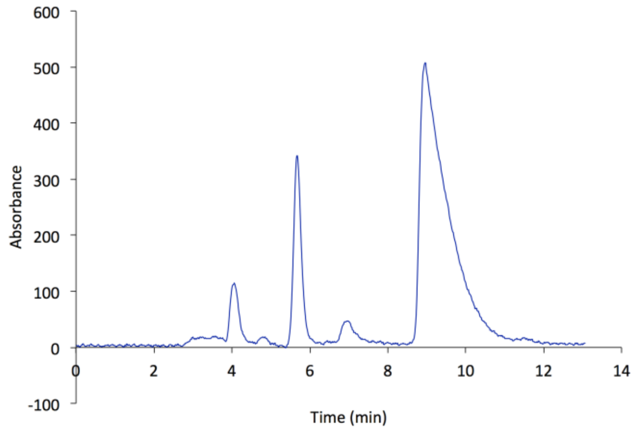

Supplementary Figure 28. HPLC separation of the esterification product from experiment 3

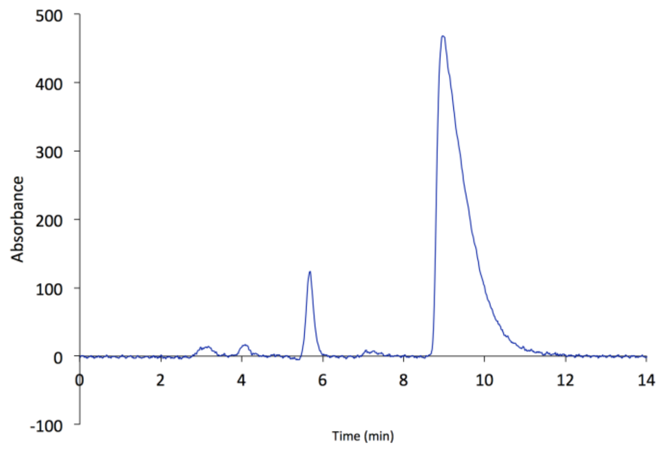

Supplementary Figure 29. HPLC separation of the esterification product from experiment 4

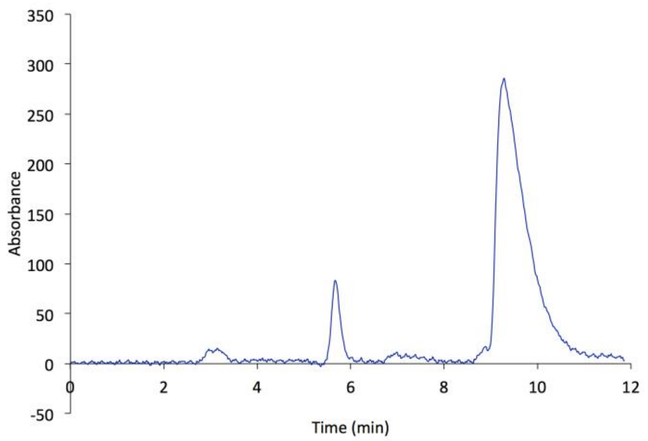

Supplementary Figure 30. HPLC separation of the esterification product from experiment 5

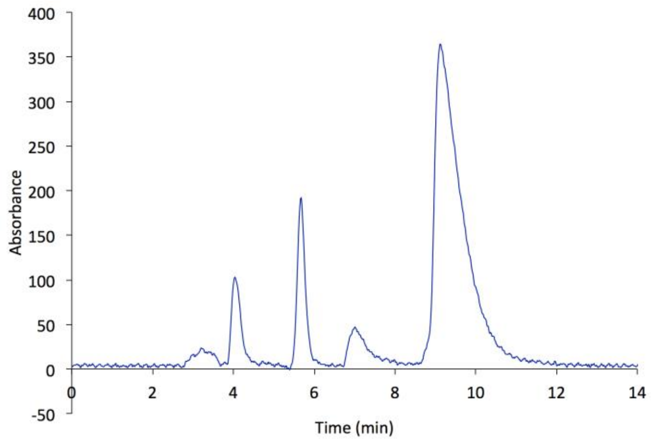

Supplementary Figure 31. HPLC separation of the esterification product from experiment 10

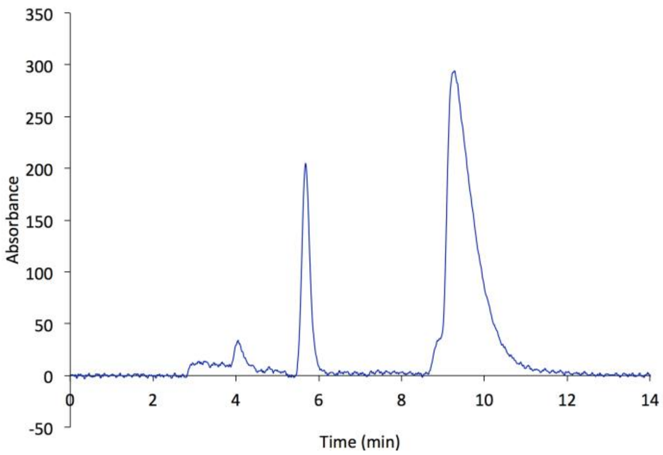

Supplementary Figure 32. ^{1}H NMR and ^{13}C NMR Spectra of **1** in ACN-d_3

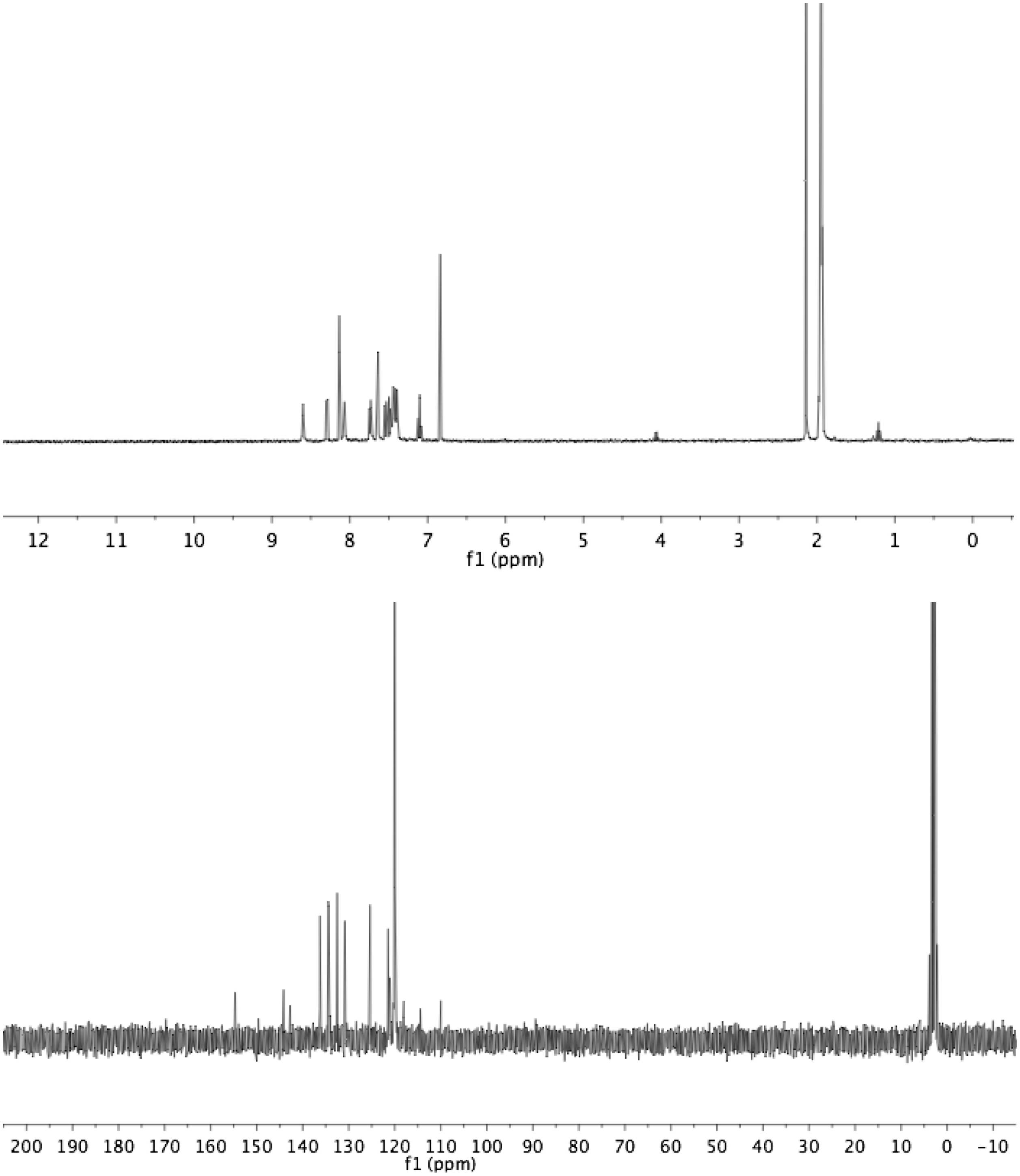

Supplementary Figure 33. ^{1}H NMR and ^{13}C NMR Spectra of **2** in ACN-d_3

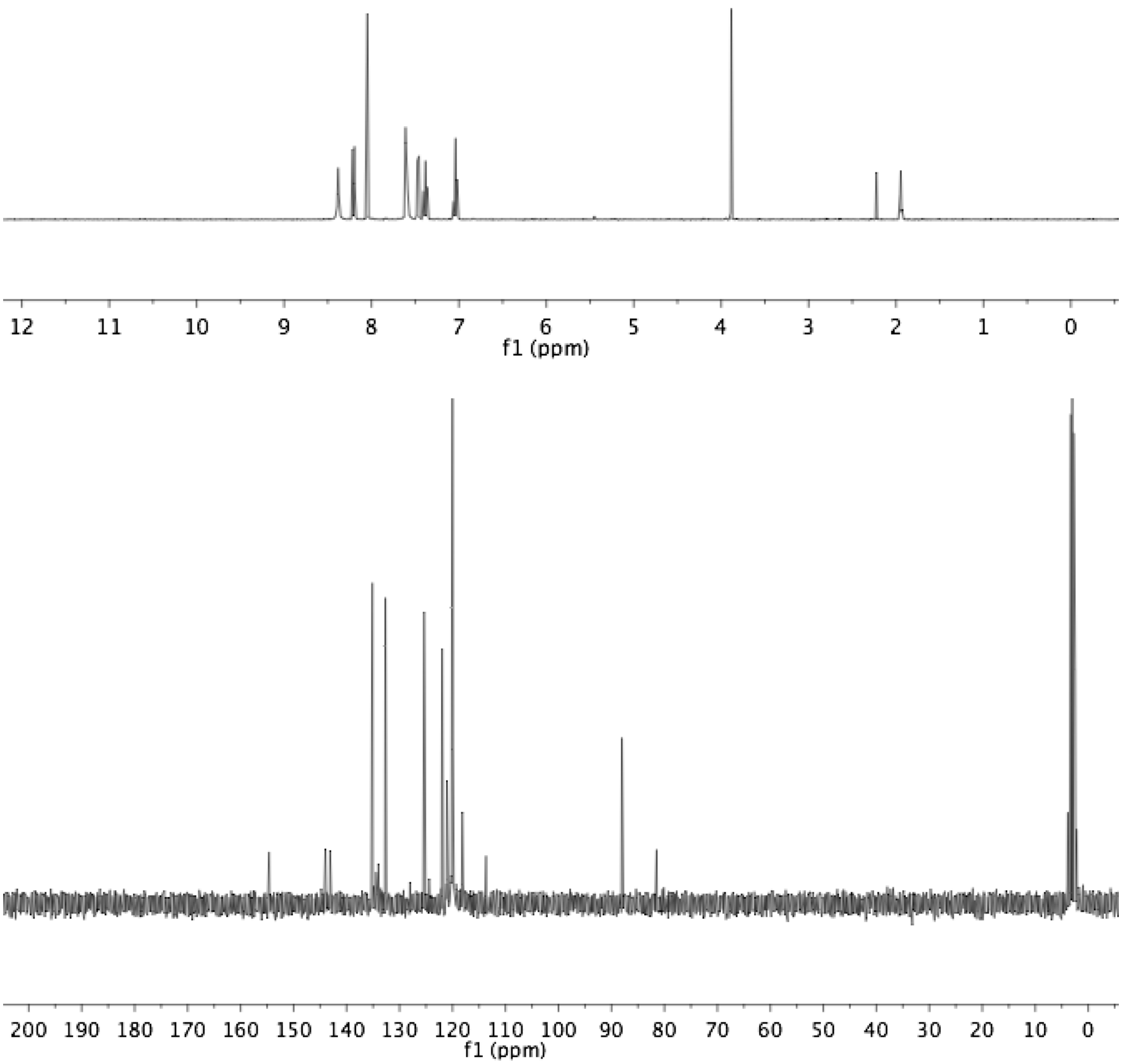

Supplementary Figure 34. ^{1}H NMR and ^{13}C NMR Spectra of **11** in ACN-d_3

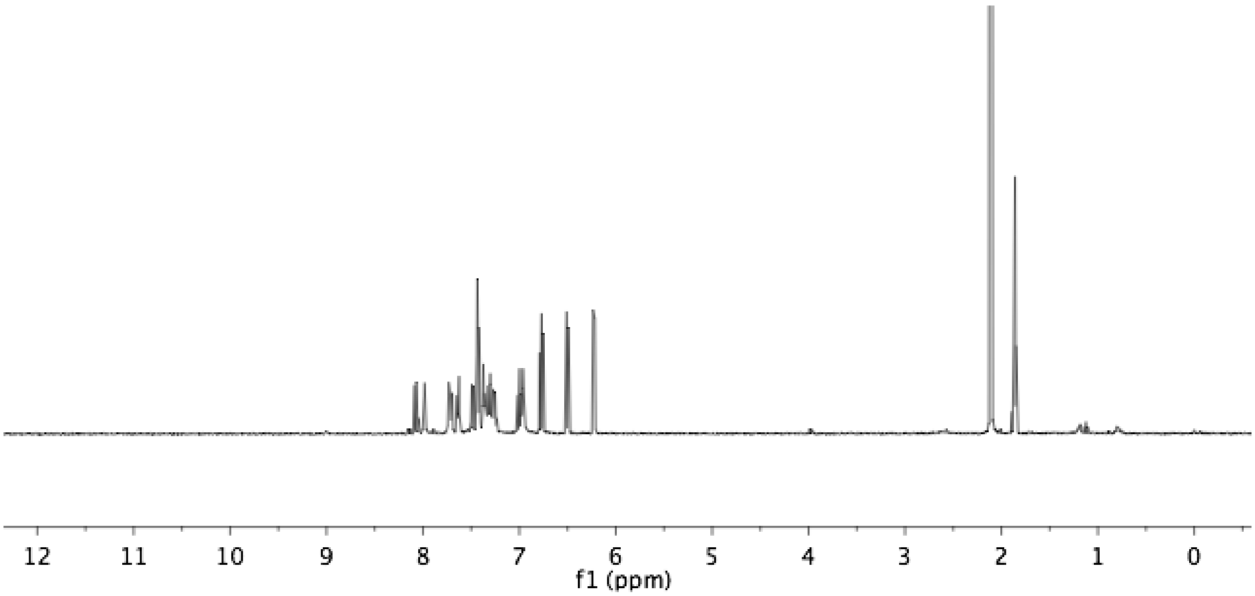

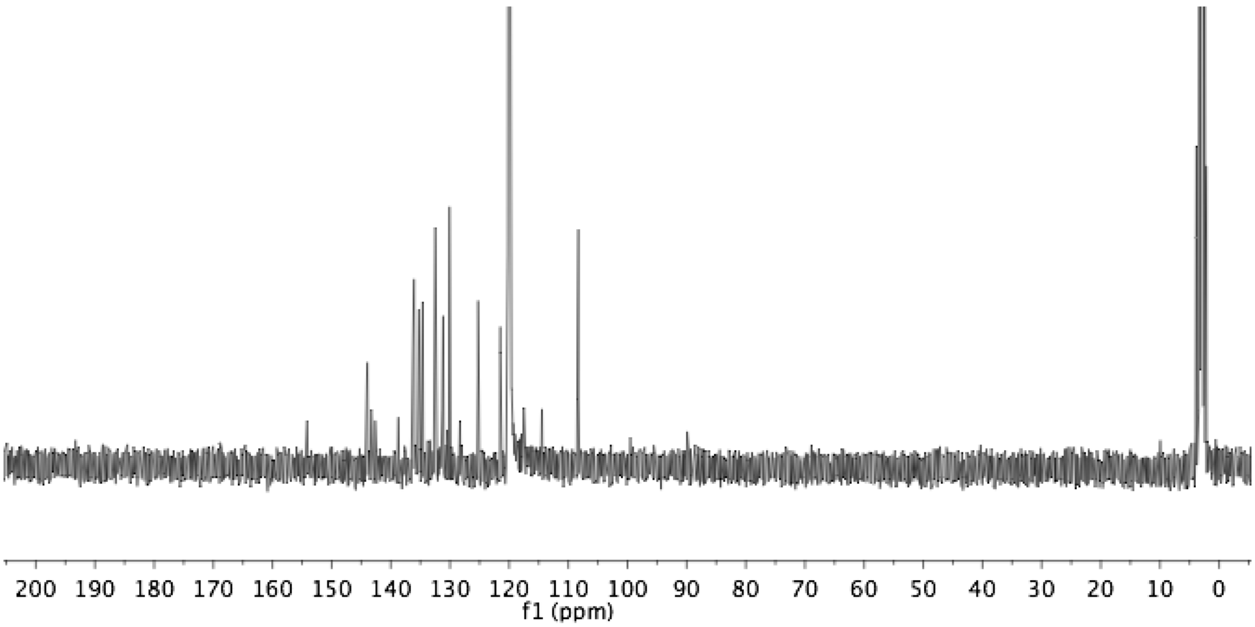

Supplementary Figure 35. X-ray structure of **2**

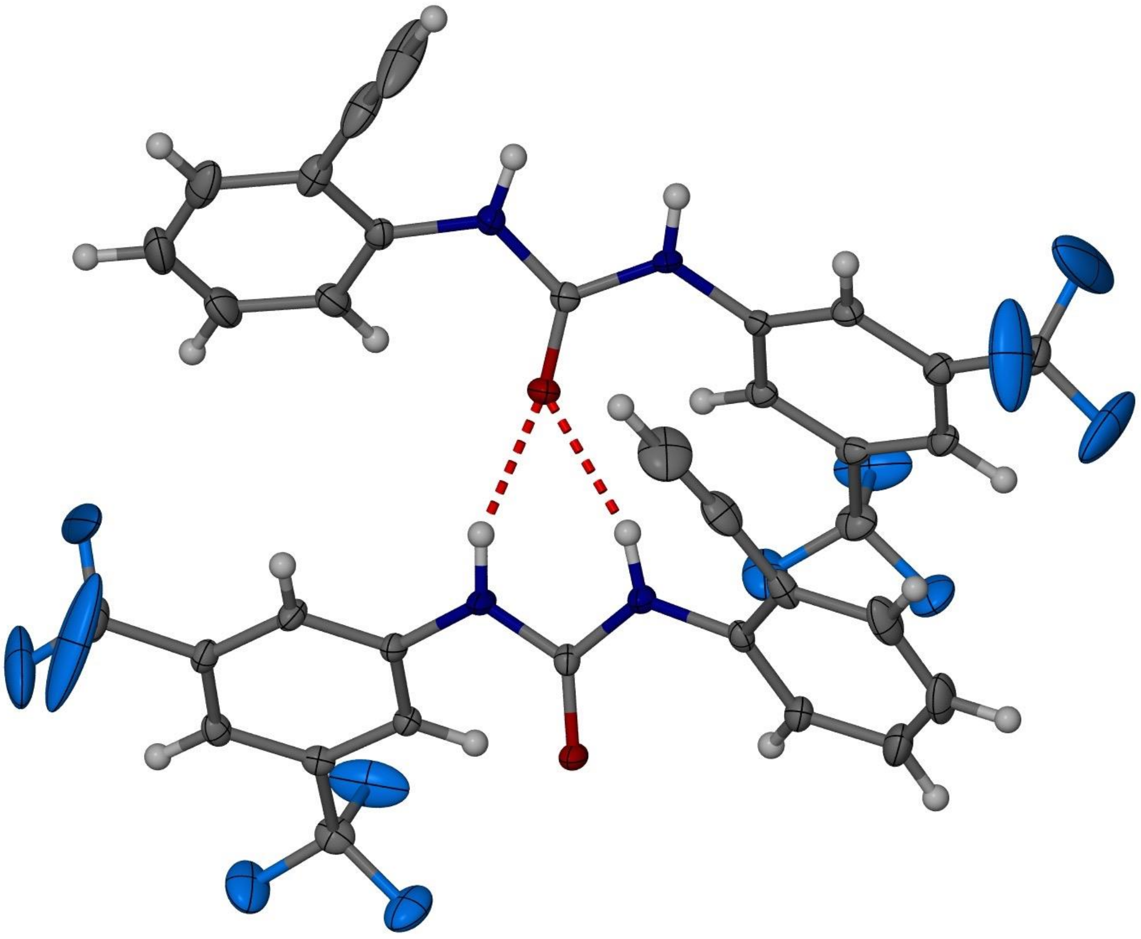

Supplementary Figure 36. X-ray structure of **1**

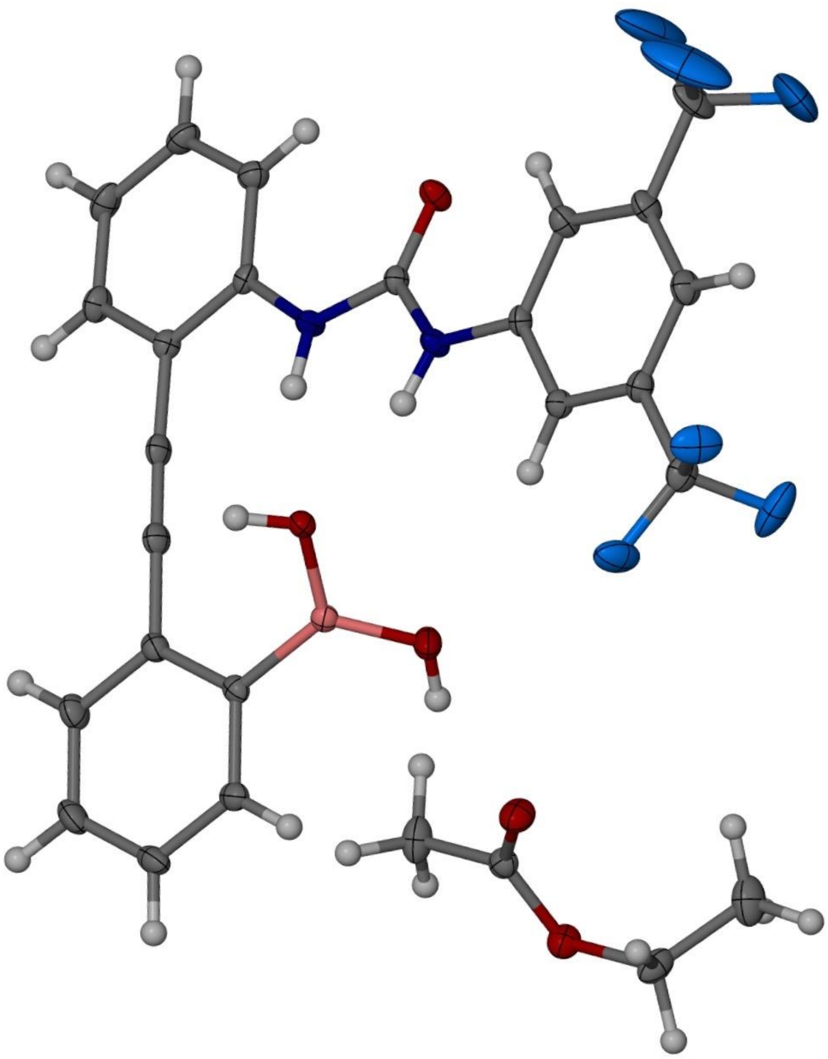

Supplementary Table 1. Experimentally determined ee of five samples of **3** using the CD responses of **1** at 300 and 330 nm.

**Actual % ee (*R*)**	**Calculated % ee at 300 nm (*R*)**	**Calculated % ee at 330 nm (*R*)**	**Average**
87.0	90.7	86.3	88.5
76.0	78.1	72.1	75.1
12.0	13.8	10.8	12.3
-26.0	-26.9	-26.7	-26.8
-68.0	-64.9	-72.7	-68.8

Supplementary Table 2. Experimentally determined concentration of five samples of **3** using the UV absorbance change of **1** at 320 and 330 nm.

**Actual Concentration (mM)**	**Calculated concentration (mM) at 320 nm**	**Calculated concentration (mM) at 330 nm**	**Average**
0.56	0.59	0.58	0.59
1.01	1.10	1.05	1.07
2.36	2.40	2.36	2.38
2.93	2.96	2.94	2.95
3.34	3.37	3.36	3.37

Supplementary Table 3. Gravimetric analysis and CD and UV measurements.

**Experiment**	**Gravimetric analysis (mg)**	**CD Intensity at 300 nm (mdeg)**	**CD Intensity at 330 nm (mdeg)**	**UV Absorbance at 320 nm (AU)**	**UV Absorbance at 330 nm (AU)**
1	8.8	14.01	10.91	1.75	1.64
2	3.8	4.63	3.69	1.54	1.34
3	2.4	4.32	3.14	1.49	1.26
4	4.6	6.69	5.67	1.56	1.39
5	1.9	1.96	1.93	1.46	1.23
10	2.1	2.29	2.02	1.48	1.25

Supplementary Table 4. Comparison of the calculated ee and concentration values of mandelic acid with the actual values determined by traditional methods.

Traditional analysis			Chiroptical sensing	
Experiment	% Ee (*S*)	Yield (%)	% Ee (*S*)	Yield (%)
1	79.3	86.6	77.1	84.8
2	67.6	37.1	61.1	34.3
3	86.4	23.2	80.9	25.0
4	83.1	45.7	76.6	40.2
5	72.5	18.6	64.4	14.0
10	64.1	21.1	58.7	17.8

Supplementary Table 5. Details of the chiroptical sensing analysis.

**Experiment**	**Calculated % yield at 320 nm**	**Calculated % yield at 330 nm**	**Average**	**Calculated % ee at 300 nm**	**Calculated % ee at 330 nm**	**Average**
1	85.4	84.2	84.8	77.2	77.0	77.1
2	33.8	34.7	34.3	61.5	60.6	61.1
3	22.3	27.6	25.0	81.2	80.5	80.9
4	40.6	39.8	40.2	77.8	75.3	76.6
5	14.7	13.2	14.0	64.1	64.7	64.4
10	17.9	17.6	17.8	58.4	59.0	58.7

Supplementary Table 6. Comparison of the analysis time and solvent consumption.

**Technique**	**Analysis Time (per reaction)**	**Solvent Use (per reaction)**
Traditional yield and ee analysis	Flash column and gravimetry: 18 min<br>Esterification: 180 min<br>Chiral HPLC: 12 min<br>**Total: 210 min**	Flash column: 100 mL<br>Esterification: 5 mL<br>Chiral HPLC: 15 mL<br>**Total: 120 mL**
Dual mode chemoensing	Sample preparation: 2 min<br>UV measurement: 0.5 min<br>CD measurement: 0.5 min<br>**Total: 3 min**	UV and CD measurements with the same sample: 3 mL<br>**Total: 3 mL**

Supplementary Methods

1. Enantioselective sensing experiments

A stock solution of sensor **1** (0.006 M) in anhydrous ACN was prepared and 500 μL portions were placed in 4 mL vials. Separately, solutions of substrates **3-9** (0.15 M) in anhydrous ACN were prepared. Et_3N (21 μL, 0.15 mmol) was added to each of the substrate solutions. Then, 20 μL (0.003 mmol) of each substrate solution was added to the solutions of **1** (Supplementary Figure 1). CD analysis was conducted immediately following the addition of the substrate using sample concentrations of 1.80 x 10^{-4} M in ACN with a standard sensitivity of 100 mdeg, a data pitch of 0.5 nm, a bandwidth of 1 nm, a scanning speed of 500 nm s^{-1} and a response of 0.5 s using a quartz cuvette (1 cm path length). The data were baseline corrected and smoothed using a binomial equation (Supplementary Figures 2-8). Control experiments with **3-9** did not show any CD signal at the wavelength of interest.

2. Quantitative ee and concentration analysis

2.1. Ee determination using mandelic acid 3

The change in the CD as a function of sample ee was investigated using samples of the complexes derived from **1** and varying ee of **3**. A stock solution of **1** (0.006 M in ACN) was prepared and 500 μL portions were placed in 4 mL vials. Into these vials, solutions of **3** (0.15 M in ACN) of varying enantiomeric composition (+100, +80, +60, +40, +20, 0, -20, -40, -60, -80, -100 %ee) were added. CD analysis was carried out as described above at 1.80 x 10^{-4} M in ACN. The Cotton effect amplitudes at 300 and 330 nm were plotted against the enantiomeric excess of **3** (Supplementary Figures 9-11).

Five solutions of **1** were prepared and **3** was added at varying enantiomeric compositions. Using the regression equation obtained above and the measured CD intensity at 300 nm and 330 nm, the ee of these samples was determined (Supplementary Table 1).

2.2. Determination of the concentration of 3

The change in the UV signature of **1** upon addition of **3** was analyzed. A stock solution of **1** (0.006 M in ACN) was prepared and 500 μL portions were placed in 4 mL vials. A stock solution of **3** (0.15 mmol in 1 mL ACN) and Et_3N (21 μL, 0.15 mmol) was also prepared. To the solutions of **1** was added **3** in varying amounts (0, 10, 20, 30, 40, 50, 60, 70, 80, 90, 100, 120, 140, 160, 180 and 200 mol%). UV spectra were collected with an average scanning time of 0.1 s, a data interval of 1nm, and a scan rate of 600 nm/min. The UV absorbance at 320 and 330 nm increased steadily upon addition of up to 1 equivalent of **3**. When the concentration of **3** was in excess of 100 mol%, the UV absorbance remained mostly constant. Plotting and curve fitting of the UV absorbance at 320 and 330 nm against the molar ratio of [**3**]/[**1**] from 0 to 100 mol% gave linear equations (Supplementary Figures 12-16).

Five solutions of **3** at varying concentrations were prepared and analyzed as described above. Using the regression equations obtained as described above and the UV absorbance at 320 and 330 nm, the concentration of these samples was determined (Supplementary Table 2).

3. MS Analysis of the sensor mandelic acid, chloromandelic acid and hexahydromandelic acid adducts

A solution of **1** (2.5 mg, 0.005 mmol), **3** (0.76 mg, 0.005 mmol) and Et_3N (0.7 μL) in 1 mL of anhydrous ACN was prepared. Electrospray mass spectrometry (negative ion mode) showed the presence of the adduct **1·3** having a 1:1 stoichiometry. The same MS spectrum was obtained when 2 equivalents of **3** were added. A solution of **1** (2.5 mg, 0.005 mmol), **5** (0.79 mg, 0.005 mmol) and Et_3N (0.7 μL) in 1 mL of anhydrous ACN was prepared. Electrospray mass spectrometry (negative ion mode) showed the presence of the adduct **1·5** having a 1:1 stoichiometric ratio. A solution of **1** (2.5 mg, 0.005 mmol), (*R*)-**3** (0.76 mg, 0.005 mmol) and NEt_3 (0.7 μL) in 1 mL of anhydrous ACN was prepared. Electrospray mass spectrometry (negative ion mode) showed the presence of the adduct of **1** and **3** having a 1:1 stoichiometry. To this mixture was then added (*R*)-**4** (0.93 mg, 0.005 mmol). ESI-MS analysis showed an additional peak at 677.7 m/z corresponding to an adduct of **1** and **4** with a 1:1 stoichiometry. No additional species were observed (Supplementary Figures 17-19).

4. NMR and CD analysis of the chemosensing mechanism

A solution of **1** (10 mg, 0.02 mmol), **3** (3.04 mg, 0.02 mmol) and Et_3N (2.8 μL, 0.02 mmol) in ACN-d_3 (0.5 mL) was subjected to ^1H and ^{11}B NMR analysis. The ^1H NMR spectra showed a strong downfield shift for the urea protons upon addition of **3** and Et_3N. The ^{11}B NMR showed an upfield shift upon addition of **3** in both the presence and absence of Et_3N. For all ^{11}B NMR spectra, a spectrum of pure ACN was subtracted to eliminate baseline noise. ^{11}B NMR analysis was also conducted using **1** with *O*-acetylmandelic acid **14** (3.95 mg, 0.02 mmol). No shift in the ^{11}B NMR was observed (Supplementary Figures 20-22).

Four samples of sensor **1** and (*R*)-**3** (1:1) were generated in anhydrous ACN. A stock solution of **1** was prepared by dissolving 11.8 mg (0.024 mmol) in 4 mL of anhydrous ACN. This stock solution was then separated into 0.5 mL (0.003 mmol) portions. To a stock solution of (*R*)-**3** (22.8 mg, 0.15 mmol in 1 mL of anhydrous ACN) were added 21 μL of Et_3N (0.15 mmol). To each 0.5 mL sensor stock solution were then added 20 μL (0.003 mmol) of the mandelic acid/Et_3N solution. To these samples were added 0.0 (blue), 0.5 (red), 1.0 (green) and 10 (purple) molar equivalents of water and CD spectra were collected at a concentration of 1.8×10^{-4} M (Supplementary Figure 23).

5. Ee and concentration analysis of mandelic acid 3 obtained by reduction of phenylglyoxylic acid 12 with (+)-DIP-Cl

Solutions of phenylglyoxylic acid, **12**, (10 mg, 0.06 mmol), an amine additive (0.06 mmol) and (+)-DIP-Cl (21.4 mg, 0.067 mmol) in 0.5 mL of anhydrous solvent were stirred in 4 mL vials under air for 12 hours at room temperature (Supplementary Figures 24 and 25). The reaction was quenched with 1M NaOH (100 μL, 0.1 mmol) and H_2O_2 (10 μL, 30% in H_2O, 0.3 mmol) and stirred for 30 minutes. 1M HCl was then added (150 μL) and the solvent was removed in vacuo. From the crude reaction mixture, 1 mg of the white solid was removed for UV and CD analysis. For traditional analysis (gravimetry and chiral HPLC) the remaining portion of the material was purified by flash chromatography on silica gel (EtOAc) to give **3** as a white solid. Each column consumed ~100 mL of solvent and required ~16 minutes, including column packing, collection,

and solvent removal. For chiral HPLC analysis, **3** was converted to the methyl ester **13** by refluxing in 5 mL of anhydrous methanol for three hours in the presence of *p*-TSA (0.1 molar equivalent). The ee of the methyl mandelate **13** was determined by HPLC on a Chiralcel OD column using hexane:*i*-PrOH (80:20 v/v) as mobile phase at 1 mL/min, $t_{1,}$ (*R*) = 5.6 min, $t_{2,}$ (*S*) = 9.2 min and required ~15 mL of solvent and ~12 minutes per sample (Supplementary Figures 26-31).

The crude solid (0.5 mg) was added to a 0.5 mL solution of the sensor (1.48 mg, 0.003 mmol) in ACN. Triethylamine (4.2 µL, 0.03 mmol) was then added. CD analysis was conducted as described above. If a CD signal was observed, subsequent UV analysis was conducted as described above. The molar ratio χ° of [**3**]/[**1**] was calculated using equations 1 and 2 derived from the calibration curves generated from the UV absorbances at 320 and 330 nm. The average value χ° was then used to determine the enantiomeric excess using equations 4 and 5, derived from the calibration curves at 300 and 330 nm. The previously determined UV and CD calibration curves were used for all analyses and new calibrations were not required. The results are listed in Supplementary Tables 3-5.

Equation 1 (UV at 320 nm):

$$\chi_{320} = \frac{A_{320} - 1.4049}{0.4027} \qquad (1)$$

Equation 2 (UV at 330 nm):

$$\chi_{330} = \frac{A_{330} - 1.1212}{0.5937} \qquad (2)$$

Equation 3 (average of the UV responses):

$$\chi^{\circ} = \frac{\chi_{320} + \chi_{330}}{2} \qquad (3)$$

Equation 4 (CD at 300 nm):

$$ee_{300} = \frac{\frac{mdeg_{300}}{\chi^{\circ}} - 1.622}{-0.193} \qquad (4)$$

Equation 5 (CD at 330 nm):

$$ee_{330} = \frac{\frac{mdeg_{330}}{\chi^{\circ}} - 0.7712}{-0.1749} \qquad (5)$$

CD sensing of the reaction mixtures of experiments 6-9 and 11-16 did not show a measurable ee and these runs were not further analyzed. The results of all other runs were analyzed by UV/CD sensing and traditional gravimetric analysis of isolated product and chiral HPLC. The direct chiroptical chemosensing of the yield, ee, and sense of asymmetric induction from 0.5 mg of the crude reaction mixture of **3** obtained by reduction of phenylglyoxylic acid with (+)-DIP-Cl

required significantly less time and solvent than gravimetry and chiral HPLC. Each optical measurement required less than 30 seconds and solvent consumption was reduced to less than 3 mL (for sample dilution) for each sample (a single sample was used for both CD and UV analysis). Importantly, the chirality sensing of the crude product mixtures eliminates elaborate purification steps as well as product derivatization for ee analysis. By contrast, the traditional analysis required purification via flash chromatography followed by gravimetric analysis and chiral HPLC of the methyl ester of **3**. This led to significantly higher solvent consumption (120 mL) and analysis time (3.5 hours) per sample (Supplementary Table 6).

6. Crystallography

A single crystal of compound **2** was obtained by slow evaporation of a concentrated ACN solution (Supplementary Figure 35). Crystallographic analysis was performed at 100 K using a Siemens platform diffractometer with graphite monochromated Mo-Kα radiation (λ = 0.71073 Å). Data were integrated and corrected using the Apex 2 program. The structure was solved by direct methods and refined with full-matrix least-square analysis using SHELX-97-2 software. Non-hydrogen atoms were refined with anisotropic displacement parameters. The asymmetric unit contains two molecules of **2**. Crystal structure data: Formula $C_{17}H_{10}F_6N_2O$, M = 372.27, crystal dimensions 0.21 x 0.20 x 0.14 mm, triclinic, space group P-1, *a* = 9.0933(4) Å, *b* = 13.1316(5) Å, *c* = 27.5087(10) Å, α = 96.726°, β = 90.011°, γ = 90.440°, V = 3262.1 $Å^3$, Z = 6, ρ_{calcd} = 1.501 g cm^{-3}.

A single crystal of **1** was obtained by slow cooling of a hot, concentrated EtOAc solution (Supplementary Figure 36). The asymmetric unit contains one molecules of **1** and one molecule of EtOAc. Crystal structure data: Formula $C_{27}H_{23}BF_6N_2O_5$, M = 580.28, crystal dimensions 0.23 x 0.16 x 0.13 mm, orthorhombic, space group $P2_12_12_1$, *a* = 7.2017(3) Å, *b* = 14.5481(7) Å, *c* = 25.1933(11) Å, α = 90°, β = 90°, γ = 90°, V = 2369.5(2) $Å^3$, Z = 4, ρ_{calcd} = 1.460 g cm^{-3}.
